# Predicting unplanned hospital visits in older home care recipients: a cross-country external validation study

**DOI:** 10.1186/s12877-021-02521-2

**Published:** 2021-10-14

**Authors:** Jet H. Klunder, Veronique Bordonis, Martijn W. Heymans, Henriëtte G. van der Roest, Anja Declercq, Jan H. Smit, Vjenka Garms-Homolova, Pálmi V. Jónsson, Harriet Finne-Soveri, Graziano Onder, Karlijn J. Joling, Otto R. Maarsingh, Hein P. J. van Hout

**Affiliations:** 1 Department of General Practice, Amsterdam University Medical Center, Vrije Universiteit, Amsterdam, The Netherlands; 2 Department of Epidemiology and Data Science, Amsterdam University Medical Center, Vrije Universiteit, Amsterdam, The Netherlands; 3 Department on Aging, Netherlands Institute of Mental Health and Addiction (Trimbos Institute), Utrecht, The Netherlands; 4 Center for Care Research & Consultancy (LUCAS) & Center for Sociological Research (CESO), KU Leuven, Leuven, Belgium; 5 Department of Psychiatry, Amsterdam Public Health Research Institute, Amsterdam University Medical Center, Vrije Universiteit, Amsterdam, The Netherlands; 6 Department of Economics and Law, HTW Berlin University of Applied Sciences, Berlin, Germany; 7 Department of Geriatrics, Landspitali University Hospital and Faculty of Medicine, University of Iceland, Reykjavík, Iceland; 8 Department of Wellbeing, National Institute for Health and Wellbeing, Helsinki, Finland; 9 Department of Cardiovascular, Endocrine-Metabolic Diseases and Aging, Istituto Superiore di Sanità, Rome, Italy; 10 Department of Medicine for Older People, Amsterdam University Medical Center, Vrije Universiteit, Amsterdam, The Netherlands

**Keywords:** Risk prediction models, Unplanned hospitalizations, Emergency department visits, Geographical validation, Home care

## Abstract

**Background:**

Accurate identification of older persons at risk of unplanned hospital visits can facilitate preventive interventions. Several risk scores have been developed to identify older adults at risk of unplanned hospital visits. It is unclear whether risk scores developed in one country, perform as well in another. This study validates seven risk scores to predict unplanned hospital admissions and emergency department (ED) visits in older home care recipients from six countries.

**Methods:**

We used the IBenC sample (*n* = 2446), a cohort of older home care recipients from six countries (Belgium, Finland, Germany, Iceland, Italy and The Netherlands) to validate four specific risk scores (DIVERT, CARS, EARLI and previous acute admissions) and three frailty indicators (CHESS, Fried Frailty Criteria and Frailty Index). Outcome measures were unplanned hospital admissions, ED visits or any unplanned hospital visits after 6 months. Missing data were handled by multiple imputation. Performance was determined by assessing calibration and discrimination (area under receiver operating characteristic curve (AUC)).

**Results:**

Risk score performance varied across countries. In Iceland, for any unplanned hospital visits DIVERT and CARS reached a fair predictive value (AUC 0.74 [0.68–0.80] and AUC 0.74 [0.67–0.80]), respectively). In Finland, DIVERT had fair performance predicting ED visits (AUC 0.72 [0.67–0.77]) and any unplanned hospital visits (AUC 0.73 [0.67–0.77]). In other countries, AUCs did not exceed 0.70.

**Conclusions:**

Geographical validation of risk scores predicting unplanned hospital visits in home care recipients showed substantial variations of poor to fair performance across countries. Unplanned hospital visits seem considerably dependent on healthcare context. Therefore, risk scores should be validated regionally before applied to practice. Future studies should focus on identification of more discriminative predictors in order to develop more accurate risk scores.

**Supplementary Information:**

The online version contains supplementary material available at 10.1186/s12877-021-02521-2.

## Background

Ageing in place policies and the reduction of nursing home beds require older adults to live increasingly longer in the community. Community-dwelling older adults are more prone to encounter accidents and suboptimal management of chronic disease [1, 2]. This consequently increases the risk of unplanned hospital use [3]. Emergency department (ED) visits and unplanned hospitalizations can negatively affect older people’s lives, e.g. causing rapid functional decline and death [4, 5].

To allow a timely intervention, several risk scores have been developed to identify older adults at risk of future ED visits or unplanned hospitalizations [6–8]. These studies however all stressed the need for external validation. Geographical validation, which validates in samples from other geographical areas, provides strong evidence on the performance and generalizability of a risk score [9]. Older populations and organization of emergency care differ between countries. An accurate validated risk score in one country, might therefore not perform as well in another. It is thus worthy to assess the performance of risk scores across different countries.

In addition, frailty is associated with higher risk for hospitalizations [10, 11]. It therefore seems reasonable to use a general frailty indicator for stratifying older patients on their risk of unplanned hospital visits. Validation of frailty indicators has been performed to predict combined adverse events (e.g. long-term care admissions, hospitalizations and death), but rarely unplanned hospital visits solely [12, 13]. Therefore, we also evaluated the validity of frailty indicators to predict unplanned hospital visits.

The objective of this study was to assess geographical validity of existing risk scores as well as of frailty indicators to predict ED visits and unplanned hospitalizations in older home care recipients from six countries.

## Methods

We reported the current study according to the Transparent Reporting of a multivariable prediction model for Individual Prognosis or Diagnosis (TRIPOD) statement [14].

### Source of data: design and sample of the IBenC study

We conducted a comparative validation study by using data of the cross-European “Identifying best practices for care-dependent elderly by Benchmarking Costs and outcomes of community care” (IBenC) study [15].

Data collection of the IBenC study was performed between January 2014 and August 2016 in six European countries: Belgium, Finland, Germany, Iceland, Italy and the Netherlands. Participants were home care recipients expected to receive care for 6 more months. Other selection criteria can be found elsewhere [15]. For this study we used data from baseline and 6 month follow-up.

### Data collection of the IBenC cohort

Data on care recipient characteristics and resource utilization were collected with the interRAI Home Care (interRAI-HC) instrument [16]. The interRAI-HC contains about 300 items, including domains of function, cognition, health, social support and service use with good to excellent interrater reliabilities [16]. Trained (research) nurses collected the data at the residences of home care recipients. All sources of data information were used: patient interviews, care files, observations and information obtained from informal and formal caregivers [15].

### Outcome measures

The dichotomous outcomes of this study were the presence of “unplanned hospital admissions”, “ED visits”, and “any unplanned hospital visits” from 3-6 months after baseline. This timeframe was defined as such, since InterRAI-HC assesses hospital admissions and ED visits 90 days prior to follow-up [17].

### Study population and loss to follow-up

At baseline, the IBenC cohort consisted of 2656 home care recipients. After 6 months, 347 participants (13.1%) were lost to follow-up (see Supplementary Figure 1, Additional File 1). Participants with missing outcomes because of death or a nursing home admission had significantly higher age, more comorbidities and more functional impairments compared to care recipients with an available outcome (data not shown). Moreover, these participants did not match the target population for which the risk scores are developed, and were therefore excluded (*n* = 210) [18]. Regarding the remaining missing data (*n* = 137), multiple imputation (MI) was applied, resulting in a total sample of 2446 cases for this study [19].

### Risk scores

We calculated seven risk scores to predict unplanned hospitalizations or ED visits in the IBenC study sample. A detailed description of the risk scores and their use within the IBenC data can be found in Additional File 2.

Four of the risk scores were developed to predict hospital admissions or ED visits in older people specifically. We selected these risk scores because of their accurate predictive value after validation and/or their applicability within the IBenC data. The risk scores are listed below:

1. Detection of Indicators and Vulnerabilities for Emergency Room Trips (DIVERT) scale [6]
  The DIVERT is a prognostic case-finding tool for ED use within 6 months. The tool was derived and internally validated using routinely collected data from interRAI-HC assessments in home healthcare recipients in Canada. Geographic (within Canada) and temporal validations by the same research group demonstrated similar performance [20]. More than 80% of home care recipients in the development cohort were aged ≥65 years. ED use was assessed through electronic records.
2. The Community Assessment Risk Screen (CARS) [7]
  The CARS is a tool developed in Illinois (USA) for stratifying community-dwelling older adults at risk for hospitalizations or ED visits within 12 months. The development cohort consisted of Medicare fee-for-service patients and the tool was externally validated in a cohort of individuals enrolled in a Medicare Risk Demonstration. All participants were aged ≥65 years. Data were obtained through telephone interviews and mailed questionnaires. Healthcare utilization was mainly determined from claims files.
3. The Emergency Admission Risk Likelihood Index (EARLI) [8]
  The EARLI is a tool to predict the likelihood of emergency hospital admission within 12 months. Data of the development and (external) validation cohorts came from questionnaires sent to older people aged ≥75 years registered with general practices in north-west England. Emergency hospitalizations were determined through administrative and clinical data.
4. The Previous Acute Admissions (PAA) score
  Prior hospital visits is considered an important predictor of unplanned hospitalizations [21, 22], and is an item in all above risk scores. Because of its predictive potential in combination with easy applicability, we decided to assess the performance of previous acute admissions (PAA) as an individual risk score. We have named this measure the PAA-score. It is a discrete measure, based on interRAI-HC data, which accumulates the number of unplanned ED visits and hospitalizations in the past 90 days.

In addition, we computed three generic frailty indicators in the IBenC data;

5) The MDS Changes in Health, End-stage disease and Symptoms and Signs (CHESS) scale [23]
  The CHESS was developed using routinely collected data and was designed to identify health instability (i.e. mortality and hospitalizations) within 30 days in long-term care residents. The development cohort consisted of Medicare beneficiaries aged 65 years and over, newly admitted to a nursing home. The scale was temporally validated in a cohort admitted 1 year later and was also tested in long-stay nursing home residents. Death and hospitalizations were obtained from medical files.
6) Fried’s Frailty Criteria (FFC) [24, 25]
  The FFC was developed to define a phenotype of frailty based on five criteria. The criteria were based on a prospective study of adults aged 65 years and over, and were validated in community-dwelling older women. For this study, we applied the Bandeen-Roche specifications to operationalize the criterion ‘Weakness’ [25].
7) The Frailty Index (FI) [26, 27]
  The FI developed by Rockwood et al. [28] is based on an accumulation of deficits approach. The FI is calculated as the proportion of potential deficits and therefore ranges from 0 to 1. For this study we combined the FI’s developed by Armstrong et al. and Lutomski et al., resulting in an FI of 44 deficits.

### Statistical analysis

Descriptive statistics were performed for baseline characteristics and main outcomes. We performed univariate logistic regression analyses with loss to follow-up as dependent variable to determine differences in prognostic factors across those lost to follow-up and those without loss to follow-up. All variables with missing data were handled through application of MI by chained equations (m = 5) (Additional File 1) [29]. We compared two MI procedures; one multilevel method, with country as cluster variable, and one normal MI method including the country variable. The dataset with multilevel imputation [29] was used as primary results.

We used the original scoring systems to compute the risk scores and used these scores to assess their performance. Performance of the risk scores was evaluated based on discrimination and calibration. Discrimination describes the ability of a risk score to differentiate between participants with and those without the outcome. This was estimated with the area under the receiver operating characteristic curve (AUC) with 95% confidence intervals [14].

Calibration reflects the agreement between observed and predicted values. Calibration was inspected graphically with calibration plots [14].

For calibration of the risk scores developed using logistic regression (i.e. CARS and EARLI), we used the coefficients reported in the original publications and determined the intercept in the IBenC data, since the intercept was not reported in the original publication. The intercept and coefficients of the PAA-score were completely based on the IBenC data. The calibration plots of these three risk scores were constructed for all three outcomes.

DIVERT and CHESS were not based on logistic regression and we therefore used the observed proportion of the outcome from the original publications (i.e. ED visits and hospital admissions, respectively) in the respective risk categories (1–6 and 0–5, respectively) as the expected proportion for that risk category within the IBenC data [30]. This could only be done for equal outcomes.

The FFC and FI did not use logistic regression nor had an identical outcome, calibration measures could therefore not be assessed.

Statistical analyses were performed with SPSS version 26.0 and R Studio Version 1.1.463. For MI and pooling analyses in R, we used R packages mice, miceadds, micemd and psfmi.

## Results

At baseline, the mean age of the complete cohort was 82.7 years, the majority was female (67.6%) and 56.7% lived alone. A child (in law) (52.4%) mainly was the primary informal caregiver, followed by the spouse in 20.4%.

Thirty-one percent had two or more comorbidities (i.e. coronary heart disease, congestive heart failure, chronic obstructive pulmonary disease (COPD), diabetes mellitus, history of stroke or cancer). On average, Italian home care recipients had the highest dependency level, while Dutch recipients were least impaired. These and more baseline data are provided in Additional File 3.

Table 1 shows the frequencies of outcomes, i.e. hospital admissions, ED visits or one of these events in the 90 days prior to follow-up, overall and separated by country. Overall, at follow-up, 510 participants (22.4%) had been admitted to the hospital at least once, 328 (14.4%) had at least one ED visit and 644 participants (28.3%) had been either hospitalized or visited the ED. Italians were more frequently admitted to the ED (33.3%) as well as to the hospital (44.2%) compared to participants from the other countries. Descriptives of the original derivation and validation cohorts of the risk scores can be found in Additional File 4. Additional File 5 lists the distributions of the risk scores per country for this study.

**Table 1:** Performance scores, reflected as pooled AUC, per country for each risk score

	Hospital admissions	ED visits	Any hospital visit
**Italy, N (%)**	220 (44.1)	166 (33.3)	259 (51.9)
**Risk score**	**Pooled AUC (95% CI)**	**Pooled AUC (95% CI)**	**Pooled AUC (95%-CI)**
DIVERT	0.57 (0.51–0.62)	0.56 (0.50–0.61)	0.58 (0.53–0.63)
CARS	0.61 (0.55–0.65)	0.58 (0.53–0.63)	0.62 (0.57–0.67)
EARLI	0.60 (0.55–0.65)	0.54 (0.48–0.59)	0.67 (0.52–0.62)
PAA-score	0.61 (0.57–0.66)	0.63 (0.58–0.68)	0.62 (0.57–0.67)
CHESS	0.57 (0.52–0.62)	0.52 (0.47–0.57)	0.57 (0.52–0.61)
FFC	0.55 (0.50–0.60)	0.51 (0.46–0.56)	0.53 (0.48–0.58)
FI	0.54 (0.49–0.59)	0.52 (0.47–0.57)	0.52 (0.47–0.57)
**the Netherlands, N (%)**	41 (17.0)	27 (11.1)	58 (23.6)
**Risk score**	**Pooled AUC (95% CI)**	**Pooled AUC (95% CI)**	**Pooled AUC (95%-CI)**
DIVERT	0.58 (0.47–0.68)	0.65 (0.53–0.75)	0.60 (0.50–0.69)
CARS	0.55 (0.43–0.65)	0.63 (0.49–0.74)	0.58 (0.48–0.67)
EARLI	0.55 (0.43–0.65)	0.60 (0.46–0.73)	0.56 (0.46–0.66)
PAA-score	0.53 (0.44–0.62)	0.54 (0.45–0.63)	0.55 (0.47–0.63)
CHESS	0.52 (0.41–0.63)	0.60 (0.48–0.71)	0.53 (0.44–0.63)
FFC	0.52 (0.42–0.62)	0.57 (0.42–0.71)	0.52 (0.43–0.60)
FI	0.56 (0.45–0.66)	0.54 (0.38–0.68)	0.56 (0.46–0.66)
**Belgium, N (%)**	66 (14.0)	15 (3.3)	71 (15.2)
**Risk score**	**Pooled AUC (95% CI)**	**Pooled AUC (95% CI)**	**Pooled AUC (95%-CI)**
DIVERT	0.68 (0.59–0.75)	0.68 (0.51–0.81)	0.67 (0.59–0.74)
CARS	0.67 (0.55–0.77)	0.68 (0.47–0.83)	0.66 (0.56–0.75)
EARLI	0.63 (0.55–0.71)	0.70 (0.51–0.84)	0.63 (0.55–0.71)
PAA-score	0.67 (0.61–0.73)	0.70 (0.55–0.81)	0.67 (0.61–0.73)
CHESS	0.57 (0.50–0.64)	0.54 (0.39–0.68)	0.57 (0.50–0.64)
FFC	0.55 (0.48–0.62)	0.57 (0.44–0.70)	0.55 (0.49–0.62)
FI	0.53 (0.45–0.60)	0.52 (0.39–0.65)	0.53 (0.46–0.60)
**Iceland, N (%)**	75 (21.0)	30 (8.4)	92 (25.8)
**Risk score (range)**	**Pooled AUC (95% CI)**	**Pooled AUC (95% CI)**	**Pooled AUC (95%-CI)**
DIVERT	0.73 (0.66–0.79)	0.67 (0.55–0.77)	0.74 (0.68–0.80)
CARS	0.72 (0.65–0.79)	0.72 (0.61–0.81)	0.74 (0.67–0.80)
EARLI	0.72 (0.65–0.79)	0.55 (0.43–0.67)	0.69 (0.61–0.75)
PAA-score	0.69 (0.63–0.75)	0.65 (0.55–0.74)	0.69 (0.63–0.75)
CHESS	0.57 (0.50–0.64)	0.58 (0.48–0.67)	0.58 (0.51–0.64)
FFC	0.58 (0.49–0.66)	0.53 (0.43–0.63)	0.56 (0.48–0.64)
FI	0.60 (0.53–0.67)	0.55 (0.45–0.65)	0.60 (0.53–0.67)
**Finland, N (%)**	87 (20.0)	85 (19.6)	142 (32.7)
**Risk score (range)**	**Pooled AUC (95% CI)**	**Pooled AUC (95% CI)**	**Pooled AUC (95%-CI)**
DIVERT	0.69 (0.62–0.75)	0.72 (0.67–0.77)	0.73 (0.67–0.77)
CARS	0.63 (0.56–0.69)	0.61 (0.54–0.67)	0.64 (0.58–0.70)
EARLI	0.62 (0.55–0.69)	0.57 (0.50–0.64)	0.60 (0.54–0.65)
PAA-score	0.66 (0.59–0.72)	0.70 (0.63–0.76)	0.69 (0.64–0.74)
CHESS	0.55 (0.48–0.61)	0.56 (0.49–0.62)	0.53 (0.48–0.59)
FFC	0.56 (0.50–0.61)	0.55 (0.49–0.61)	0.55 (0.50–0.61)
FI	0.61 (0.54–0.68)	0.54 (0.47–0.61)	0.58 (0.52–0.64)
**Germany, N (%)**	51 (11.5)	19 (4.3)	63 (14.2)
**Risk score (range)**	**Pooled AUC (95% CI)**	**Pooled AUC (95% CI)**	**Pooled AUC (95%-CI)**
DIVERT	0.64 (0.55–0.72)	0.52 (0.38–0.66)	0.62 (0.54–0.70)
CARS	0.59 (0.50–0.68)	0.54 (0.40–0.68)	0.59 (0.51–0.67)
EARLI	0.65 (0.56–0.73)	0.59 (0.45–0.72)	0.65 (0.57–0.72)
PAA-score	0.61 (0.54–0.68)	0.55 (0.44–0.66)	0.61 (0.54–0.67)
CHESS	0.59 (0.50–0.67)	0.53 (0.40–0.65)	0.56 (0.48–0.63)
FFC	0.54 (0.45–0.61)	0.58 (0.44–0.71)	0.51 (0.43–0.58)
FI	0.51 (0.42–0.60)	0.52 (0.38–0.66)	0.52 (0.43–0.60)

Abbreviations: *95%* CI 95% confidence interval; *AUC* area under the curve; *CARS* Community Assessment Risk Screen; *CHESS MDS* Changes in Health, End-stage disease and Symptoms and Signs; *DIVERT* Detection of Indicators and Vulnerabilities for Emergency Room Trips; *EARLI* Emergency Admission Risk Likelihood Index; *ED* emergency department; *FFC* Fried’s frailty criteria; *FI* frailty index; *PAA* previous acute admissions. Number of imputed outcomes per country [unplanned admissions - ED visits - any unplanned hospital visit]: Italy [3 - 2 - 3]; Netherlands [48 - 47 - 48]; Belgium [38 - 36 - 38]; Iceland [15 - 15 - 15]; Finland [20 - 20 - 20]; Germany [46 - 46 - 46]

**Fig. 1:**
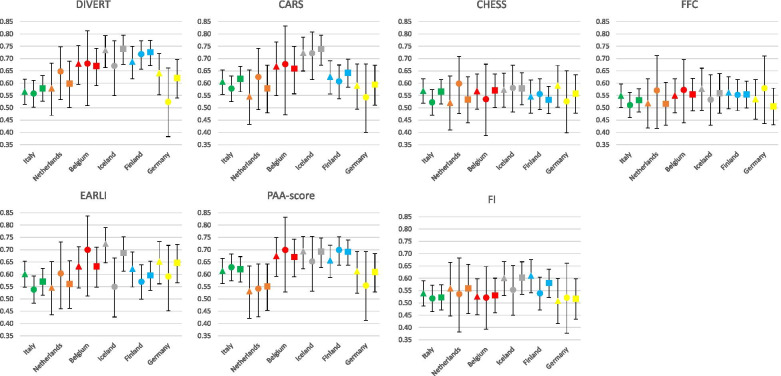
Predictive value of the risk scores per country and outcome. Predictive values of the seven risk scores per country expressed as pooled area under the curve with 95%-confidence intervals. Outcomes are presented as hospital admission (▲), emergency department visit (●) and any hospital visit (■), respectively. Abbreviations: *AUC* Area Under the Curve; *CARS* Community Assessment Risk Screen; *CHESS* MDS Changes in Health, End-stage disease and Symptoms and Signs; *DIVERT* Detection of Indicators and Vulnerabilities for Emergency Room Trips; *EARLI* Emergency Admission Risk Likelihood Index; *FFC* Fried’s frailty criteria; *FI* frailty index; *PAA* previous acute admissions

The performance of the risk scores are shown in Table 1 and Fig. 1. In Iceland, DIVERT, CARS and EARLI reached a fair predictive value (AUC of 0.73, 0.72 and 0.72, respectively for the outcome hospital admissions). DIVERT had fair performance in Finland for ED visits and the combination of ED visits and admissions (AUC of 0.72 and 0.73, respectively). Regarding the prediction of any unplanned hospital admission or ED visit, DIVERT performed best across all countries, except for Italy. In Italy, CARS and the PAA-score performed best. AUCs of the frailty indicators did not exceed 0.6 on any outcome.

In general, the specific risk scores reached higher AUCs than the frailty indicators.

Calibration of the models was moderate to poor (see Additional File 6). Unfortunately, for some analyses of EARLI and CARS in the German and Belgian populations, there was too little variance in probabilities, which made grouping impossible and calibration plots could therefore not be created.

## Discussion

### Summary of results

In this external validation study, we found substantial variation in predictive performance between the risk scores and between the six countries. Overall, risk scores showed poor to fair discrimination and calibration. In Iceland, DIVERT, CARS and EARLI reached fair predictive values for unplanned hospital admissions. In Finland, DIVERT had fair performance predicting ED visits and any unplanned hospital visits. In other countries, AUCs did not exceed 0.70. The specific risk scores (i.e. DIVERT, CARS, EARLI and PAA-score) performed better than the generic frailty indicators (i.e. CHESS, FFC and FI).

Prediction models should be externally validated in new, but comparable samples before they can be applied in practice [31]. Validations in more homogenous samples, or samples with different case-mix compared to the development sample, often result in worse discriminative performance. As shown in Additional Files 3 and 4, the IBenC sample differed considerably from the development samples of the original studies. Moreover, CARS and EARLI were developed in community-dwelling older adults, whether or not they were receiving home care. And even though DIVERT was developed in home care recipients as well, Supplemental Table 6 shows the IBenC sample differed from the home care recipients from the DIVERT cohort. For example, mean age of the IBenC cohort was 7 years older and more participants within the IBenC cohort lived alone. Because of the differences in case-mix and amount of care received between the IBenC cohort and the development cohorts of CARS, EARLI and DIVERT, predictive performances of the development studies and this study cannot be directly compared. This study should thus be interpreted as a test of transportability of these models to patients from different source populations (i.e. home care recipients from different countries), than as a test of (statistical) reproducibility. In conclusion, we emphasize that our results can only be generalized to older home care recipients from these countries, and not to older community-dwellers in general.

Not unexpectedly, performance of the risk scores differed across the six IBenC subsamples as well. This is partly attributable to case-mix differences between the IBenC samples. For instance, the Italian sample was very different compared to the other countries (e.g. few recipients living alone, and high prevalence of ≥2 comorbidities). Still, healthcare context probably has affected performances too. Hospitalizations and ED visits are very dependent on the availability of other healthcare services, for example early consultation and better monitoring may prevent unplanned hospital visits [32, 33]. These outcome measures therefore depend on local healthcare policy, accessibility and availability. This strongly pleas for validation of risk scores in the area of use, and, if possible, to consider adding national or regional system variables to internationally validated risk scores.

Our results are similar to those found in other validation studies in community-dwelling older adults [20, 34, 35]. Geographical and temporal validation of the DIVERT showed similar performance predicting ED visits (i.e. AUCs between 0.62 and 0.65) in four Canadian regions as the original cohort (AUC 0.62 [6]) [20]. Remarkably, the DIVERT model was more accurate predicting ED visits in Finnish and Icelandic home care recipients (AUC 0.72 and 0.67, respectively).

AUCs of 0.6–0.7 are not uncommon for predictions of ED visits and unplanned hospital admissions [21, 36]. However, one can argue whether a validated risk score with, at best, moderate performance is of added value to a clinician’s decision making and will improve quality of patient care through timely preventive interventions.

Previous hospital admissions and cardiorespiratory diseases and symptoms are predominantly present variables in these risk scores and have shown to be important predictors of future hospital visits in other studies too [37, 38]. Predictors concerning social context and accessibility of primary care, have been marginally assessed in these risk scores, even though they have shown to be associated with healthcare utilization [39–42]. For instance, social deprivation and decreased socio-economic status are associated with increased ED attendance and unplanned hospital admissions [33, 40]. Regarding primary care, longer opening hours, more appointments slots and continuity of care have shown to reduce unplanned hospital visits [33]. These nonmedical factors could add substantial discriminative power in identifying older adults at risk of hospitalization.

### Strengths and limitations

This is a thorough geographical validation study on seven risk scores to predict unplanned hospital admissions and ED visits in six countries using MI. In general, external validation in “different but related” individuals is an essential step in the development of a prediction model, because it provides valuable information about the generalizability of the model. This step is however often skipped [9, 31]. Our study adds substantial evidence to the limited amount of external validation studies regarding the prediction of unplanned hospital visits in older home care recipients.

The IBenC cohort is a multinational cohort, which adequately reflects characteristics of older home care recipients in these countries [15]. The international component makes the data valuable to compare characteristics of home care recipients from different geographical samples. Dependency levels in the IBenC sample closely reflect previously reported dependency levels among home care recipients in several European countries including Italy, the Netherlands, Iceland, Finland and Germany [43]. However, national representativeness remains uncertain.

A selection bias may have occurred in the Italian and Dutch samples. The Italian data were retrieved retrospectively on routine care recipients with 6 months follow-up. Therefore this sample may overrepresent persons receiving home care for a longer period. Nonetheless, a ceiling effect in distribution of the risk scores in the Italian sample did not occur (see Additional File 5). In the Netherlands, one of the main reasons provided for refusal to participate was cognitive impairment, therefore the proportion of patients with cognitive impairment was low. However, of the specific risk scores, only EARLI is dependent on cognitive function and this would therefore not have affected our results to a great extent.

We emphasize that the IBenC sample is very different in characteristics and care received from the general older population. Determinants of hospitalization in this population might differ from general populations and therefore, the results of this study are not transferable to the general older population.

Because the risk scores were developed to be applicable in living persons that were not too vulnerable, we decided to omit recipients with missing outcomes because of death or admission to a nursing home. As a consequence, we excluded vulnerable patients with a conceivably high probability of unplanned hospital visits. This may have influenced predictive performance. However, these participants covered less than 10% of the total sample size and would probably not have substantially affected our results.

Next, for some items in the risk scores, the original items were not directly transferable into interRAI-HC items (e.g weakness in FFC). We calculated the risk scores based on reasonable proxy items, some items might therefore not fully cover the original items. In addition, for weakness in FFC we used a validated and interRAI specified adjustment, which is an acceptable proxy [25].

Lastly, outcome measures were assessed 6 months after baseline with a 3 month recall period. We therefore missed outcomes from the first 3 months after baseline. In addition, CARS and EARLI were developed to predict the outcome within 12 months. Since risk changes over time, particularly for the prediction of unplanned hospital visits, predictions within a shorter time span might be more accurate [44, 45]. These factors might have influenced the performance of the risk scores.

## Conclusions

Geographical validation of multiple risk scores for unplanned hospital visits in home care recipients from six European countries showed poor to fair performance. Unplanned hospitalizations depend, at least partly, on local healthcare policy. Therefore, whenever possible, risk scores should be validated regionally before applied in practice. Further studies are needed to identify and compose predictors and risk scores better able to predict unplanned hospital visits, preferably in diverse care contexts. Early identification of patients at high risk of unplanned hospital visits may prompt healthcare professionals to attempt targeted interventions, such as targeted patient education, intensive monitoring or integrated management for specific conditions or patient groups [46, 47].

## Supplementary Information

**Additional file 1.** : Loss to follow-up and elaboration on multiple imputation methodology. This file shows the flow of the IBenC cohort for this article and how missing data was handled. It also elaborates on the MI method, such as the methods used for imputation and selected key variables.

**Additional file 2.** : Comprehensive description of risk scores. This file elaborates on how the risk scores assessed in this article were developed.

**Additional file 3.** : Baseline characteristics of the IBenC data. This table provides information on the baseline characteristics of each country subcohort within the IBenC study

**Additional file 4.** : Characteristics of the cohorts from the original studies and the IBenC cohort. These tables compare the characteristics of the cohorts of the original studies with the characteristics of the IBenC data. They provide a global insight in the differences in case-mix between these cohorts.

**Additional file 5.** : Distribution of the risk scores. Descriptive statistics of each of the seven risk scores per country

**Additional file 6.** : Calibration plots. Calibration plots of the risk scores for each country, provided this could be assessed.

## Data Availability

The datasets used and analyzed during the current study are available from the corresponding author on reasonable request.

## Abbreviations

AUC: Area under the receiver operating characteristic curve
CARS: Community Assessment Risk Screen
CHESS: MDS Changes in Health, End-stage disease and Symptoms and Signs
DIVERT: Detection of Indicators and Vulnerabilities for Emergency Room Trips
EARLI: Emergency Admission Risk Likelihood Index
ED: Emergency department
FFC: Fried’s Frailty Criteria
FI: Frailty Index
IBenC: Identifying best practices for care-dependent elderly by Benchmarking Costs and outcomes of community care study
InterRAI-HC: InterRAI-Home Care instrument
MI: Multiple imputation
PAA-score: Previous Acute Admissions score
