# Supplementary material for: Predicting unplanned hospital visits in older home care recipients: a cross-country external validation study
Source: BMC Geriatr. 2021 Oct 14;21:551. doi: 10.1186/s12877-021-02521-2 (PMC8515741; doi:10.1186/s12877-021-02521-2)
Supplement: Supplementary file 1 — Additional file 1. : Loss to follow-up and elaboration on multiple imputation methodology. This file shows the flow of the IBenC cohort for this article and how missing data was handled. It also elaborates on the MI method, such as the methods used for imputation and selected key variables. [file 12877_2021_2521_MOESM1_ESM.docx]

## **Additional file 1 Loss to follow-up and elaboration on multiple imputation methodology**

**Supplementary Figure 1 Flow of the IBenC cohort for current study and handling of missing data**

We compared the AUC’s of two multiple imputation methods; one multilevel method and one normal imputation method. For the multilevel method, country was set as cluster variable. The descriptions of the different methods are shown in Table A7.

**Supplementary Table 1 Description of different imputation methods**

|  | **Dichotomous variables**  **(R package)** | **Categorical and scale variables**  **(R package)** |
| --- | --- | --- |
| **Normal imputation** | logreg (mice) | pmm (mice) |
| **Multilevel imputation** | 2l.bin (mice) | 2l.pmm (miceadds) |

logreg = logistic regression, pmm = predictive mean matching, 2l.bin = two-level logistic model, 2l.pmm = two-level predictive mean matching. An elaboration of these methods can be found elsewhere.(12)

To create variable specific imputation models containing a maximum of 30 predictors per imputed variable, we included variables with a minimum correlation coefficient value of 0.27. We selected 23 key variables that were included in the imputation models: (i.e. outcome measures (n=2), variables needed for the risk scores with over 10% missing values (n=11) and variables concerning home environment (n=5) and informal caregiver of the participants(n=5)). The last two categories of key variables were selected based on literature or, if not available, on clinical expertise. If a predictor variable had >20% missing data or was irrelevant to the imputed variable (based on clinical expertise or common sense), the predictor was excluded.

To check the imputation runs, we made convergence plots. We plotted the means and standard deviations of each iteration number in convergence plots, which showed healthy convergence. Healthy convergence is diagnosed when the variance between the imputation chains is almost equal to the variance within the chains. Consequently, we created strip plots to visualize observed and imputed data, which showed no large differences between imputed and observed values. Strip and convergence plots were evaluated by two authors (JK & MH). Strip and convergence plots can be requested from the author.

**Supplementary Table 2 Key variables used for multiple imputation**

| **Outcome variables** |
| --- |
| Inpatient acute hospital with overnight stay: # times past 90 days |
| Emergency room visit (not counting overnight stay): # times past 90 days |
| **Variables with >10% missing** |
| Number of prescribed medications |
| Stroke/CVA |
| Coronary heart disease |
| COPD |
| Congestive heart failure |
| Pneumonia |
| Urinary tract infection |
| Cancer |
| Diabetes |
| Timed walk |
| Distance walked |
| **Variables concerning home environment** |
| Person or relative feels that person would be better of living elsewhere |
| Home health aides |
| In the last 3 days # of days went out of the house |
| Major life stressors in the last 90 days |
| Disrepair of the home |
| **Variables concerning informal caregiver** |
| Informal helper relationship |
| Living arrangement |
| Informal helper is unable to continue in caring activities |

CVA, cerebrovascular accident; COPD, chronic obstructive pulmonary disease
