# Supplementary material for: Predicting unplanned hospital visits in older home care recipients: a cross-country external validation study
Source: BMC Geriatr. 2021 Oct 14;21:551. doi: 10.1186/s12877-021-02521-2 (PMC8515741; doi:10.1186/s12877-021-02521-2)
Supplement: Supplementary file 2 — Additional file 2. : Comprehensive description of risk scores. This file elaborates on how the risk scores assessed in this article were developed. [file 12877_2021_2521_MOESM2_ESM.docx]

## **Additional file 2 Comprehensive description of the risk scores**

**Supplementary Figure 2 Composition of DIVERT scale**

**Detection of Indicators and Vulnerabilities for Emergency Room Trips (DIVERT)^1^**

DIVERT was developed using a population based retrospective cohort study of routine interRAI-HC assessments linked to ED records. The study was executed among 361,942 older Canadians who were expected to receive home care services for at least 60 days. Ninety-five RAI HC 2.0 items were used to select potential predictive variables. One or more ED visits within 6 months was used as the dependent variable. The cohort was randomly split in a derivation (75%) and validation cohort (25%). A recursive partitioning method was used to derive the DIVERT Scale informed by a multinational clinical panel. Figure 1 shows the included variables within the DIVERT scale. Scores range from 1 to 6, the higher the score, the greater the risk of ED admission. The validation sample achieved an AUC of 0.62 (95% CI: 0.61-0.62).

**Supplementary Figure 3 Composition of CARS**

**Community Assessment Risk Screen (CARS)^2^**

CARS was designed to identify community dwelling older adults at high risk for ED admission or hospitalization within 12 months. The study population consisted of a development cohort of 411 Medicare fee-for-service patients and an external validation cohort of 1,054 individuals enrolled in a Medicare Risk Demonstration. Both were recruited in Illinois, USA. All included patients were 65 years or older and survived for 12 more months after enrolment. They were interviewed by telephone and mail questionnaire to acquire baseline data on demographics, health status and healthcare utilization. Outcome measures came from claims files, except for ED use in the development cohort, which was based on self-report. Backward stepwise logistic regression was used to identify significant variables in the development cohort that predicted the dependent variable. Three variables were identified, as shown in Figure 2.

Depending on the answers, the scores can range from 0 to 9. A cut off-score of 4 was used to define a low- and high risk category. Using this cut-off, the AUC of the derivation sample was 0.74, in the validation sample the AUC reached 0.67 (no 95%-CI’s reported).

As shown in figure 2, CARS uses “any hospital use in prior year” as independent variable, within the IBenC sample only data of the prior 90 days were available, this may have influenced our results.

**Supplementary Figure 4 Composition of EARLI**

The terms in bold are the original items of the EARLI risk score, the terms not in bold are the items used in this study’s dataset.

**Emergency Admission Risk Likelihood Index (EARLI)^3^**

EARLI was developed for predicting the risk of emergency admission to hospital of community-dwelling older people aged 75 years and above. It was based on a prospective cohort study of 3,032 older adults registered with 17 general practices in Halton, a health district covering the towns of Runcorn and Widnes in northwest England. The main outcome was one or more emergency hospital admissions within 12 months, obtained through electronic health records. Candidate predictors came from a previously developed 20-item binary questionnaire. First, univariate associations between the candidate predictor variables and the emergency admission outcome were assessed. Predictors with significant associations were then fitted in a multivariable logistic regression model using stepwise forward selection. The model was developed and validated in two ways; first, the model was developed in the full dataset and internally validated using bootstrap validation with stepwise modelling. This retrieved an AUC of 0.695 (95% CI 0.671-0.719) in the development set and an AUC of 0.67 (no 95%-CI’s reported) in the bootstrapped sample. Second, the model was developed using data from Runcorn and externally validated in the Widnes sample. This external validation achieved an AUC of 0.669 (95% CI 0.630-0.709), AUC of the development set was not reported. The second procedure obtained the same predictors as the development procedure in the full dataset.

EARLI contains six questions. Scores range from 1 to 29. A starting score of 10 was chosen in order to avoid the possibility of a negative score. Score are categorized into low (1-10), moderate (11-15), high (16-20) and very high (21-29) risk.

Regarding the question ‘Can you get out of house without help’, we used ‘Locomotion independency’ as a proxy-item within the IBenC data. Anyone who needed limited assistance or more (iG2f >2) was given a score of 0. Also ‘Confusion’ was not a specific item in the IBenC data, we therefore disregarded this item and only assessed items regarding memory problems. Lastly, IBenC has data of hospitalizations 3 months prior, whereas EARLI uses hospitalization of 12 months earlier.

**Supplementary Figure 5 Composition of CHESS scale**

**Changes in Health, End-stage disease and Symptoms and Signs (CHESS)^4^**

The CHESS scale is a composite measure designed to identify health instability (i.e. adverse health outcomes, including death) in long-term care populations using routinely collected data. The scale was developed in a population of Medicare beneficiaries aged 65 and older newly admitted to a nursing home (N=1,297,117) in 2012 in the USA. The participants received an assessment using the Minimum Data Set (MDS) 3.0 in the first year of admission. The MDS 3.0 contains information on the care of residents, such as medical diagnoses and cognitive and physical functioning. Candidate predictors were items identified from the MDS 3.0 as related to the likelihood of death, based on literature. The primary outcome measure was death at 30, 60, 365 and 730 days after assessment, obtained through Medicare Master Beneficiary Summary Files.

The CHESS was validated in two cohorts, the primary cohort consisted of newly admitted residents admitted in 2013 (N=1,217,008), with identical inclusion criteria as the development cohort. The secondary validation cohort consisted of long-stay nursing home residents, who had been admitted for at least 90 days (N=1,070,672). For the primary validation cohort, hospitalization within 30 days of the assessment was assessed as outcome as well. Bivariate cox proportional hazard models were used to identify relevant candidate predictors. Hazard ratios less than 1.5 were excluded. Scores range from 0 (most stable) to 5 (least stable). The health conditions in the first step contribute a maximum of 2 points. AUC’s were not reported. In the original study, having a score of 3 or 4 was associated with greater risk of hospitalization than a score of 0 or 5.

The original item ‘food or fluid decrease’ was replaced by items ‘fluid output exceeds input’ and ‘weight loss’ within the IBenC database.

**Supplementary Table 3 Composition of Fried’s Frailty Criteria**

| **Fried’s frailty criteria and score**  **when answered ‘Yes’** | **Items in interRAI-HC** |
| --- | --- |
| Shrinking (+1) | Either, BMI < 20kg/m^2^ |
|  | Or, weight loss > 5% in last 30 days or ≥ 10%  in last 180 days |
| Weakness (+1) | Either, walked < 50 feet |
|  | Or, fully dependent walk |
|  | Or, full dependent locomotion on unit |
|  | Or, unable to balance without assistance |
|  | Or, unsteady balance or needs balance support  to balance sitting |
| Exhaustion (+1) | Either, dyspnea at rest or during day to day activities |
|  | Or, unable to finish daily activities |
| Slowness (+1) | Timed walk 4 meters in seconds  (stratified for gender and height) |
|  |  |
|  |  |
| Low activity (+1) | Either, did not have any physical activity or exercise in the last 3 days |
|  | Or, distance walked is nil |

**Fried’s frailty criteria (FFC) ^5, 6^**

FFC was developed by Fried et al. to define a phenotype of frailty in older adults.^7^ The study used data from the Cardiovascular Health Study (CHS), a prospective observational study of 5,317 older adults, aged 65 and over, with up to 7 years follow-up. The study used 5 criteria as suggested by prior research and clinical consensus to define frailty and operationalized these in the CHS. Using cox proportional hazard models frailty was associated with falls, worsening disability, hospitalization and death. This definition was validated in the Women’s Health and Aging Studies by Bandeen-Roche et al., where they found comparable results and stated the definition is applicable across diverse population samples. For our validation study, we used the measures as operationalized by Bandeen-Roche et al. (with adjustment of the criterion weakness, see below). Proxy-items within RAI instruments were checked for internal consistency in the STRIVE report.^6^

The five criteria are shown in Table 2, scores range from 0 to 5. Patients are defined frail when 3 or more criteria are met, patients scoring 2 or 3 are defined as pre-frail and patients scoring 0 to 1 as non-frail.

Weakness was originally measured through assessment of grip strength. In accordance with the STRIVE report, the criterion weakness was positive when one or more of the following criteria within the IBenC data were met; distance walked was no more than 50 feet (15.24 meter), the patient was fully dependent walking or fully dependent for locomotion on a unit, the patient was unable to balance standing without assistance, or the patient had an unsteady gait.

**Supplementary Table 4 Composition of Frailty Index**

| **Subgroup** | **Items and coding of frailty index in interRAI-HC and MDS-TOPICS** | **Weights** |
| --- | --- | --- |
| **ADL** | Eating Toilet use  Bathing  Hygiene Bed mobility Dressing upper body  Dressing lower body Walking | 0. Independent=0  1. Setup help only=0.165  2. Supervision=0.335  3. Limited assistance=0.505  4. Extensive assistance=0.67  5. Maximal assistance=0.835  6. Total dependence=1 |
| **iADL** | Walking stairs  Shopping  Housecleaning  Meal preparation  Medication management  Financial management  Transportation | 0. Independent=0  1. Setup help only=0.165  2. Supervision=0.335  3. Limited assistance=0.505  4. Extensive assistance=0.67  5. Maximal assistance=0.835  6. Total dependence=1 |
| **Health status** | Self-reported health | 0. Excellent=0  1. Good=0.33  2. Fair=0.66  3. Poor=1 |
|  | Sadness Little interest/ pleasure | 0. Not in last 3 days=0  1. But often feels that way=0.33  2. In 1-2 of last 3 days=0.66  3. Daily in last 3 days=1 |
|  | Anhedonia  Repetitive health complaints  Pain | 0. Not present=0  1. Present, not exhibited last 3 days=0.33  2. Exhibited 1-2 days of 3 days=0.66  3. Exhibited daily in last 3 days=1 |
|  | Dyspnea | 0. Absence of symptom=0  1. Present while moderate act=0.33  2. Present while normal day-to-day=0.66  3. Present at rest=1 |
|  | Dizziness  Unsteady gait | 0. Not present=0  1. Present, not exhibited last 3 days=0.25  2. Exhibited on 1 of last 3 days=0.50  3. Exhibited on 2 of last 3 days=0.75  4. Exhibited daily in last 3 days=1 |
|  | Hearing (with use of aid)  Vision (with use of aid) | 0. Adequate=0  1. Minimal difficulty=0.25  2. Moderate difficulty=0.50  3. Severe difficulty=0.75  4. No hearing/vision=1 |
|  | Bladder continence | 0. Continent=0  1. Control with any catheter or ostomy over last 3 days=0.2  2. Infrequently incontinent, not in last 3 days=0.4  3. Occasionally incontinent, less than daily=0.6  4. Frequently incontinent daily, but some control present=0.8  5. Incontinent, no control present=1 |
|  | Weight loss (≥ 5 percent in the last 30 days, or ≥ 10 percent in the last 180 days) | 0. No=0  1. Yes=1 |
|  | Activity level/hours exercise | None=1  Less than 1 hour=0.75  1-2 hours=0.5  3-4 hours=0.25  More than 4 hours=0 |
|  | Cognitive skills: daily decision making | 0. Independent=0  1. Modified independence=0.25  2. Minimally impaired=0.5  3. Moderately impaired=0.75  4. Severely impaired=1 |
|  | Procedural memory | 0. Memory OK=0  1. Memory problem=1 |
|  | Fatigue | 0. None  1. Minimal Diminished energy but normal activities=0.25  2. Moderate Due to diminished energy, unable to finish normal activities=0.5  3. Severe Due to diminished energy, unable to start normal activities=0.75  4. Unable to commence any normal activities=1 |
|  | Distance walked | 0. Did not walk=1  1. Less 5 meters (15 feet)=0.8  2. 5-49 meters (15-149 feet)=0.6  3. 50-99 meters (150-299 feet)=0.4  4. 100+ meters (300+ feet)=0.2  5. 1+ kilometres (1/2 mile or more)=0 |
|  | 4 meter walk - time in seconds | 1 ≤ time ≤ 5 = 0  time = 6 = 0.2  7 ≤ time ≤ 9 = 0.4  10 ≤ time ≤ 14 = 0.6  15 ≤ time ≤ 20 = 0.8  Time >30 = 1  If wheelchair or bedridden = 1 |
|  | Falls | 0. No falls in last 90 days = 0  1. No fall in last 30 days, but fell 31-90 days ago = 0.33  2. One fall in last 30 days = 0.66  3. Two or more falls in last 30 days = 1 |
|  | BMI | BMI ≤18 = 1  BMI ≥30 = 1  BMI >18.5 & BMI <30 = 0 |
| **Diagnoses** | Cancer | 0. Not present = 0  1. Present = 1 |
|  | Diabetes mellitus |  |
|  | COPD |  |
|  | Coronary heart disease |  |
|  | Congestive heart failure |  |
|  | Stroke/CVA |  |
| **Social activity** | Change in social activities | 0. No decline = 0  1. Decline, not distressed = 0.1  2. Decline, distressed = 1 |
| **Frailty Index** | add up all deficit values divided by number of items | |

**Frailty index (FI)^8, 9^**

The FI developed by Rockwood et al. is based on an accumulation of deficits approach.^10^ The FI is calculated as the proportion of potential deficits that are present in a given individual. For this study we calculated the operationalization of an FI by Armstrong et al..^8^ Armstrong et al. constructed an FI of 50 deficits based on interRAI HC data using procedures outlined in Searle et al.^11^. They used a database (n=23,952) of home care clients (aged 65+) of eight Community Care Access Centres in Ontario, Canada. Data were collected using interRAI HC. Multivariate cox analysis revealed this FI to be a significant predictor of institutionalization and death.

Since the IBenC study used a different (suite) version of the interRAI HC. We derived an FI combining the FI version of Armstrong with items from MDS-TOPICS (The Older Persons and Informal Caregivers Survey), as validated elsewhere.^9^ This FI counted 44 deficits. These deficits included items regarding ADL/iADL status, health status, diagnoses and social activities. Each individual deficit is weighed according to the weights provided in the third column of Supplementary Table 2. Since FI is composed of a proportion of deficits, scores range from 0 to 1. Rockwood et al. and Armstrong et al. both found that the maximum score that could be achieved was around 0.66.^8, 10^

**References**

1. Costa, AP, Hirdes, JP, Bell, CM, et al. Derivation and validation of the detection of indicators and vulnerabilities for emergency room trips scale for classifying the risk of emergency department use in frail community-dwelling older adults. Journal of the American Geriatrics Society 2015;63(4):763-769.

2. Shelton, P, Sager, MA, Schraeder, C. The community assessment risk screen (CARS): identifying elderly persons at risk for hospitalization or emergency department visit. The American journal of managed care 2000;6(8):925-933.

3. Lyon, D, Lancaster, GA, Taylor, S, et al. Predicting the likelihood of emergency admission to hospital of older people: development and validation of the Emergency Admission Risk Likelihood Index (EARLI). Family practice 2007;24(2):158-167.

4. Ogarek, JA, McCreedy, EM, Thomas, KS, et al. Minimum Data Set Changes in Health, End-Stage Disease and Symptoms and Signs Scale: A Revised Measure to Predict Mortality in Nursing Home Residents. Journal of the American Geriatrics Society 2018;66(5):976-981.

5. Bandeen-Roche, K, Xue, QL, Ferrucci, L, et al. Phenotype of frailty: characterization in the women's health and aging studies. The journals of gerontology Series A, Biological sciences and medical sciences 2006;61(3):262-266.

6. STRIVE Phase II Final Report. 2011:81-83.

7. Fried, LP, Tangen, CM, Walston, J, et al. Frailty in older adults: evidence for a phenotype. The journals of gerontology Series A, Biological sciences and medical sciences 2001;56(3):M146-156.

8. Armstrong, JJ, Stolee, P, Hirdes, JP, et al. Examining three frailty conceptualizations in their ability to predict negative outcomes for home-care clients. Age and ageing 2010;39(6):755-758.

9. Lutomski, JE, Baars, MA, van Kempen, JA, et al. Validation of a frailty index from the older persons and informal caregivers survey minimum data set. Journal of the American Geriatrics Society 2013;61(9):1625-1627.

10. Rockwood, K, Andrew, M, Mitnitski, A. A Comparison of Two Approaches to Measuring Frailty in Elderly People. The Journals of Gerontology: Series A 2007;62(7):738-743.

11. Searle, SD, Mitnitski, A, Gahbauer, EA, et al. A standard procedure for creating a frailty index. BMC geriatrics 2008;8:24.
