# Supplementary material for: Predicting unplanned hospital visits in older home care recipients: a cross-country external validation study
Source: BMC Geriatr. 2021 Oct 14;21:551. doi: 10.1186/s12877-021-02521-2 (PMC8515741; doi:10.1186/s12877-021-02521-2)
Supplement: Supplementary file 3 — Additional file 3. : Baseline characteristics of the IBenC data. This table provides information on the baseline characteristics of each country subcohort within the IBenC study [file 12877_2021_2521_MOESM3_ESM.docx]

**Supplementary Table 5 Baseline characteristics of the IBenC cohort**

| **Demographics** | **N (Valid %)**  **N = 2446** | **N**  **missing** | **Italy**  **N=499 (20.4)** | | **Netherlands**  **N=244 (10.0)** | | **Belgium**  **N=469 (19.2)** | | **Iceland**  **N=357 (14.6)** | | **Finland**  **N=434 (17.7)** | | **Germany**  **N=443 (18.1)** | |
| --- | --- | --- | --- | --- | --- | --- | --- | --- | --- | --- | --- | --- | --- | --- |
| Age, mean ± SD | 82.7 ± 7.4 | 0 | 81.8 ± 7.9 |  | 81.7 ± 7.40 |  | 82.3 ± 6.8 |  | 83.5 ± 7.0 |  | 82.9 ± 7.0 |  | 83.93±7.7 |  |
| Female | 1652 (67.6) | 2 | 286 (57.3) |  | 178 (73.0) |  | 319 (68.3) | 2 | 256 (71.7) |  | 296 (68.2) |  | 317 (71.6) |  |
| Living alone | 1382 (56.7) | 10 | 82 (16.4) |  | 179 (73.4) |  | 225 (49.0) | 10 | 220 (61.6) |  | 350 (80.6) |  | 326 (73.6) |  |
| **Diseases present** | | | | | | | | | | | | | | |
| Dementia (any type) | 573 (27.0) | 322 | 81 (40.9) | 301 | 12 (4.9) |  | 72 (16.0) | 18 | 72 (20.2) |  | 180 (41.5) |  | 156 (35.5) | 3 |
| Coronary heart disease | 536 (25.2) | 321 | 75 (37.9) | 301 | 40 (16.4) |  | 79 (17.5) | 17 | 138 (38.7) |  | 136 (31.3) |  | 68 (15.5) | 3 |
| Congestive heart failure | 564 (26.5) | 320 | 49 (24.7) | 301 | 70 (28.7) |  | 176 (38.9) | 16 | 105 (29.4) |  | 95 (21.9) |  | 69 (15.7) | 3 |
| Chronic Obstructive Pulmonary Disease | 221 (10.4) | 317 | 44 (22.2) | 301 | 47 (19.3) |  | 30 (6.6) | 13 | 56 (15.7) |  | 24 (5.5) |  | 20 (4.5) | 3 |
| Cancer | 220 (10.4) | 321 | 25 (12.6) | 301 | 40 (16.4) |  | 27 (6.0) | 17 | 39 (10.9) |  | 42 (9.7) |  | 47 (10.7) | 3 |
| Diabetes Mellitus | 543 (25.6) | 324 | 44 (22.3) | 302 | 72 (29.5) |  | 97 (21.6) | 19 | 54 (15.1) |  | 137 (31.6) |  | 139 (31.6) | 3 |
| ≥ 2 comorbidities* | 660 (31.1) | 327 | 78 (39.6) | 302 | 87 (35.7) |  | 122 (27.3) | 22 | 130 (36.4) |  | 136 (31.3) |  | 107 (24.3) | 3 |
| ≥ 5 prescribed medication | 1166 (63.1) | 599 | 291 (63.4) | 40 | 144 (62.3) | 13 | N/A | 469 | 301 (84.3) |  | 180 (41.8) | 3 | 250 (67.8) | 74 |
| **Functional status** | | | | | | | | | | | | | | |
| Any falls in last 90 days | 553 (22.7) | 7 | 156 (31.3) |  | 62 (25.4) |  | 123 (26.6) | 7 | 65 (18.2) |  | 92 (21.2) |  | 55 (12.4) |  |
| Bladder incontinence or use of urinary catheter/collection device | 1448 (59.3) | 5 | 357 (71.5) |  | 124 (50.8) |  | 387 (83.4) | 5 | 172 (48.2) |  | 172(39.6) |  | 236 (53.3) |  |
| Person or relative feels that person would be better of living  elsewhere | 200 (8.2) | 10 | 22 (4.4) |  | 32 (13.1) |  | 26 (5.7) | 10 | 55 (8.7) |  | 28 (6.5) |  | 37 (8.4) |  |
| **Informal caregiver** | | | | | | | | | | | | | | |
| No informal caregiver | 312 (13.0) | 41 | 3 (0.6) |  | 46 (18.9) |  | 0 (0) | 41 | 2 (0.6) |  | 76 (17.5) |  | 185 (41.8) |  |
| Informal caregiver is unable to continue in caring activities | 192 (9.0) | 303 | 58 (11.6) |  | 22 (9.0) |  | 68 (15.9) | 40 | 15 (4.2) | 2 | 10 (2.8) | 76 | 19 (7.4) | 185 |
| Primary informal caregiver expresses feelings of distress, anger or  depression | 284 (13.3) | 303 | 75 (15.2) |  | 20 (8.2) |  | 50 (11.7) | 40 | 98 (27.6) | 2 | 21 (5.9) | 76 | 19 (7.4) | 185 |
| **Unplanned hospital visits, once or more, 90 days prior to baseline** | | | | | | | | | | | | | | |
| Emergency department visits | 374 (15.4) | 23 | 210 (42.1) |  | 20 (8.2) |  | 14 (3.1) | 23 | 20 (5.6) |  | 84 (19.4) |  | 26 (5.9) |  |
| Hospital admissions | 544 (22.4) | 16 | 237 (47.5) |  | 25 (10.2) |  | 53 (11.7) | 16 | 71 (19.9) |  | 98 (22.6) |  | 60 (13.5) |  |
| Any unplanned hospital visit | 687 (28.3) | 20 | 287 (57.5) |  | 37 (15.2) |  | 59 (12.6) | 20 | 84 (23.5) |  | 145 (33.4) |  | 75 (16.9) |  |

Baseline characteristics are shown for the complete case analysis with number of missing values per item. Values are provided as N, (%) unless stated otherwise.

* Any of the following comorbidities: coronary heart disease, congestive heart failure, chronic obstructive pulmonary disease, diabetes, history of stroke or cancer
