# Supplementary material for: Predicting unplanned hospital visits in older home care recipients: a cross-country external validation study
Source: BMC Geriatr. 2021 Oct 14;21:551. doi: 10.1186/s12877-021-02521-2 (PMC8515741; doi:10.1186/s12877-021-02521-2)
Supplement: Supplementary file 4 — Additional file 4. : Characteristics of the cohorts from the original studies and the IBenC cohort. These tables compare the characteristics of the cohorts of the original studies with the characteristics of the IBenC data. They provide a global insight in the differences in case-mix between these cohorts. [file 12877_2021_2521_MOESM4_ESM.docx]

## **Additional file 4 Characteristics of the cohorts from the original studies and the IBenC cohort**

Unfortunately, descriptive information of FFC and FI was not provided, neither was the descriptive information of the internal validation cohort of the DIVERT scale.

**Supplementary Table 6 Characteristics of derivation cohort of DIVERT compared to the IBenC cohort**

|  | **DIVERT** | **IBenC cohort** |
| --- | --- | --- |
|  | Derivation  (N = 242,651) | External validation  (N = 2446) |
| Age (mean, SD) | 76.2 ± 14.1 | 82.72 ± 7.37 |
| Female | 64.2 | 67.5 |
| Living alone | 26.9 | 56.6 |
| No informal caregiver | 32.0 | 13.0 |
| Caregiver expresses distress* | 16.9 | 12.8 |
| ADL decline | 42.8 | 24.1 |
| Mood symptoms† | 36.3 | 44.2 |
| ADL impairment‡ | 31.1 | 60.9 |
| Dyspnea§ | 23.8 | 13.7 |
| Poor self-reported health | 18.1 | 18.4 |
| Cognitive impairment\|\| | 9.1 | 18.9 |
| Weight loss** | 8.2 | 9.1 |
| Cardiovascular disease†† | 42.0 | 48.8 |
| Diabetes | 24.5 | 25.7 |
| Dementia | 17.5 | 27.6 |
| COPD | 16.5 | 10.7 |
| Cancer | 15.8 | 10.4 |
| Infection‡‡ | 7.3 | 10.6 |
| Prior ED visits§§ | 19.3 | 15.3 |
| Prior hospital admissions§§ | 31.4 | 22.3 |
| Outcome: ED visits\|\|\|\| | 41.2 | 14.0 |

Values are presented as percentages, unless stated otherwise. ED, emergency department

* Primary caregiver expresses feelings of distress, anger or depression

† Based on interRAI Depression Rating Scale (DRS) ≥ 1

‡ Based on interRAI ADL Hierarchy Scale ≥ 1

§ Dyspnea at rest or when performing normal day-to-day activities

|| Based on interRAI Cognitive Performance Scale ≥ 3

** Weight loss of ≥5% in last 30 days or ≥10% in last 180 days

†† In DIVERT; stroke, congestive heart failure, coronary artery disease, or peripheral vascular disease. In IBenC peripheral vascular disease was not available and therefore not included in this composite measure.

‡‡ In DIVERT; pneumonia, tuberculosis, or urinary tract infection. In IBenC tuberculosis was not available and therefore not included in this composite measure.

§§ Hospital use in prior 90 days

|||| In DIVERT in next 6 months. In IBenC in 90 days prior to 6-month follow-up.

**Supplementary Table 7 Characteristics of derivation and validation cohorts of CARS compared to the IBenC sample**

|  | **CARS** | | **IBenC** |
| --- | --- | --- | --- |
|  | Derivation  (N = 411) | Internal validation  (N = 1054) | External validation  (N = 2446) |
| Age (mean, SD) | 75.4 ± 6.4 | 74.6 ± 6.5 | 82.72 ± 7.37 |
| Female | 75 | 56 | 68 |
| Living alone | 48 | 27 | 57 |
| ≥2 comorbidities* | 24 | 11 | 28 |
| ≥5 prescription medications | 32 | 12 | 64 |
| Prior hospital admissions† | 21 | 9 | 22 |
| Prior ED visits† | 18 | 10 | 15 |
| Any prior ED visit or hospital admission† | 29 | 14 | 28 |
| Outcome: hospital admission‡ | 21 | 17 | 22 |
| Outcome: ED visit‡ | 20 | 18 | 14 |
| Outcome: any hospital admission or ED visit‡ | 32 | 29 | 28 |

Values are presented as percentages, unless stated otherwise

* Any of the following: heart disease, diabetes, myocardial infarction, stroke, COPD, and cancer

† Any prior ED visits 6 months before baseline, any prior hospitalizations 1 year before baseline. In IBenC in the last 90 days

‡ Outcomes were measured 12 months after baseline. In IBenC in the last 90 days

**Supplementary Table 8 Characteristics of the complete EARLI cohort**

|  | **EARLI** | **IBenC cohort** |
| --- | --- | --- |
|  | **Complete cohort (N=3032)*** | **External validation (N=2446)** |
| Diabetes | 12.7 | 25.7 |
| Lung or breathing problems† | 33.3 | 33.6 |
| Heart problems‡ | 35.3 | 40.7 |
| Stroke | 13.8 | 14.7 |
| Cancer | 8.5 | 10.4 |
| Depression | 17.9 | 15.7 |
| Bladder/water problems§ | 40.1 | 59.8 |
| Leg ulcers\|\| | 7.5 | 25.0 |
| Lives alone | 45.5 | 56.7 |
| Get out of the house without help** | 74.9 | 29.7 |
| Bath or shower without help†† | 73.5 | 74.3 |
| Eyesight interferes with life‡‡ | 25.5 | 42.2 |
| Problems with memory | 28.0 | 25.2 |
| Flu vaccination last winter | 71.3 | 67.8 |
| Regularly take four or more medicines | 55.4 | 71.2 |
| Emergency admission in the last 12 months\|\|\|\| | 18.6 | 22.3 |
| Fallen to floor within last 3 months | 16.5 | 22.7 |
| Bereavement within last 6 months*** | 9.6 | 14.5 |
| State of health is good | 68.8 | 15.9 |

*N differs for each variable because of missing values

† In IBenC defined as either COPD or dyspnea at rest or when performing normal day-to-day activities

‡ In IBenC defined as either congestive heart failure or coronary artery disease

§ In IBenC defined as either urine incontinence or using a urinary collection device

|| In IBenC defined as presence of pressure ulcer or other skin ulcer

** In IBenC defined as locomotive independency

†† In IBenC defined as needing limited assistance or more

‡‡ In IBenC defined as having minimal difficulty to see in adequate light or worse

|||| In IBenC any emergency admission in last 90 days

*** In IBenC defined as major life stressors in last 90 days

**Supplementary Table 9 Characteristics of derivation and primary internal validation cohort of CHESS compared to the IBenC cohort**

|  | **CHESS** | | **IBenC** |
| --- | --- | --- | --- |
|  | Derivation  (N = 1,297,117) | Primary validation  (N = 1,217,008) | External validation  (N = 2,446) |
| Age (mean, SD) | 81.6 ± 8.2 | 81.7 ± 8.2 | 82.72 ± 7.37 |
| Female | 64.3 | 64.7 | 67.5 |
| Life expectancy < 6 months | 1.0 | 1.1 | 1.8 |
| Cognitive impairment* | 3.6 | 3.3 | 9.6 |
| Acute mental status change | 2.2 | 1.7 | 8.0 |
| Aggressive behavior† | 2.5 | 2.4 | 2.5 |
| Impaired daily decision making | 7.5 | 6.7 | 19.2 |
| Severe physical impairment‡ | 18.4 | 17.6 | 15.0 |
| Dehydration | 0.4 | 0.4 | 11.1 |
| Pressure ulcers | 4.7 | 3.4 | 2.1 |
| Swallowing disorder§ | 5.6 | 5.1 | 9.3 |
| Shortness of breath\|\| | 19.5 | 18.9 | 13.7 |

Values are presented as percentages, unless stated otherwise.

* In CHESS based on Cognitive Function Scale (CFS) Score 4. CFS 4 is comparable with interRAI CPS ≥ 5 (13)

† Based on interRAI Aggressive Behavior Score ≥ 3

‡ Based on Activity of Daily Living Scale ≥ 21

§ In CHESS based on “choking, holding food, lost liquid, or painful swallowing”. In IBenC defined as “requires diet modification to swallow solid food”

|| In rest or with exertion
