# Supplementary material for: Predicting unplanned hospital visits in older home care recipients: a cross-country external validation study
Source: BMC Geriatr. 2021 Oct 14;21:551. doi: 10.1186/s12877-021-02521-2 (PMC8515741; doi:10.1186/s12877-021-02521-2)
Supplement: Supplementary file 5 — Additional file 5. : Distribution of the risk scores. Descriptive statistics of each of the seven risk scores per country [file 12877_2021_2521_MOESM5_ESM.docx]

## **Additional file 5 Distribution of the risk scores**

**Supplementary Table 10 Distribution of the seven risk scores within each country**

|  |  | **Italy** | **Netherlands** | **Belgium** | **Iceland** | **Finland** | **Germany** |
| --- | --- | --- | --- | --- | --- | --- | --- |
| **DIVERT** | **1** | 7.2 | 12.7 | 22.9 | 22.1 | 26.3 | 39.5 |
| **(1-6)** | **2** | 26.7 | 35.2 | 33.0 | 23.8 | 30.0 | 29.9 |
|  | **3** | 19.9 | 27.9 | 26.3 | 25.8 | 15.0 | 16.8 |
|  | **4** | 21.0 | 18.0 | 6.1 | 14.6 | 8.1 | 9.3 |
|  | **5** | 16.6 | 4.1 | 3.8 | 10.1 | 13.1 | 3.4 |
|  | **6** | 8.6 | 2.0 | 7.9 | 3.6 | 7.6 | 1.1 |
|  | **Median** | 3 | 3 | 2 | 3 | 2 | 2 |
| **CARS** | **0** | 11.9 | 25.8 | 22.5 | 11.8 | 30.2 | 22.0 |
| **(0-9)** | **2** | 6.3 | 7.3 | 8.1 | 0.8 | 9.4 | 4.7 |
|  | **3** | 15.5 | 30.7 | 41.4 | 41.2 | 17.2 | 42.0 |
|  | **4** | 11.9 | 3.3 | 3.5 | 1.7 | 12.0 | 4.1 |
|  | **5** | 8.7 | 21.0 | 14.9 | 22.7 | 9.7 | 14.4 |
|  | **6** | 6.1 | 1.2 | 1.7 | 1.4 | 6.5 | 1.4 |
|  | **7** | 23.0 | 4.5 | 4.8 | 9.0 | 9.2 | 7.6 |
|  | **9** | 16.5 | 6.1 | 3.0 | 11.5 | 5.8 | 3.8 |
|  | **Median** | 5 | 3 | 3 | 3 | 3 | 3 |
| **EARLI** | **Median** | 16 | 10 | 12 | 13 | 14 | 13 |
| **(1-29)** | **25th perc** | 12 | 10 | 9 | 10 | 10 | 10 |
|  | **75th perc** | 20 | 14 | 14 | 17 | 17 | 17 |
|  | **Minimum** | 1 | 5 | 1 | 5 | 5 | 1 |
|  | **Maximum** | 29 | 29 | 29 | 29 | 29 | 26 |
| **PAA score** | **Median** | 1 | 0 | 0 | 0 | 0 | 0 |
| **(0-90)** | **25th perc** | 0 | 0 | 0 | 0 | 0 | 0 |
|  | **75th perc** | 2 | 0 | 0 | 0 | 1 | 0 |
|  | **Minimum** | 0 | 0 | 0 | 0 | 0 | 0 |
|  | **Maximum** | 10 | 4 | 42 | 5 | 13 | 6 |
| **CHESS** | **0** | 22.7 | 17.2 | 34.8 | 29.7 | 55.3 | 60.5 |
| **(0-5)** | **1** | 28 | 33.2 | 32.1 | 37.5 | 25.8 | 23.3 |
|  | **2** | 22.6 | 35.7 | 26.5 | 24.9 | 14.3 | 12 |
|  | **3** | 17.8 | 13.5 | 5.7 | 6.7 | 4.1 | 2.5 |
|  | **4** | 8.3 | 0.4 | 0.9 | 1.1 | 0.5 | 1.8 |
|  | **5** | 0.6 | 0 | 0 | 0 | 0 | 0 |
|  | **Median** | 1 | 1 | 1 | 1 | 0 | 0 |
| **FFC** | **0** | 0 | 0 | 0 | 0.1 | 5.7 | 0.3 |
| **(0-5)** | **1** | 0.1 | 13.5 | 14.1 | 17.9 | 26.5 | 17.2 |
|  | **2** | 5.7 | 38.5 | 24.8 | 26.9 | 40.6 | 34.2 |
|  | **3** | 39.9 | 33.2 | 35.9 | 38.5 | 22.7 | 33.6 |
|  | **4** | 39.3 | 12.7 | 22.4 | 15.9 | 4.6 | 12.4 |
|  | **5** | 15 | 2 | 2.8 | 0.7 | 0 | 2.3 |
|  | **Median** | 4 | 2 | 3 | 3 | 2 | 2 |
|  |  |  |  |  |  |  |  |
|  |  | **Italy** | **Netherlands** | **Belgium** | **Iceland** | **Finland** | **Germany** |
| **FI** | **Median** | 0.51 | 0.29 | 0.42 | 0.31 | 0.28 | 0.39 |
| **(0-1)** | **25th perc** | 0.43 | 0.22 | 0.34 | 0.24 | 0.22 | 0.29 |
|  | **75th perc** | 0.57 | 0.36 | 0.50 | 0.36 | 0.37 | 0.48 |
|  | **Minimum** | 0.11 | 0.06 | 0.09 | 0.09 | 0.05 | 0.05 |
|  | **Maximum** | 0.72 | 0.64 | 0.8 | 0.59 | 0.65 | 0.65 |

Except for median, 25th and 75th percentiles, minimum and maximum, values are presented as percentages. perc, percentile
