# Supplementary material for: Predicting unplanned hospital visits in older home care recipients: a cross-country external validation study
Source: BMC Geriatr. 2021 Oct 14;21:551. doi: 10.1186/s12877-021-02521-2 (PMC8515741; doi:10.1186/s12877-021-02521-2)

## **Additional file 6 Calibration plots**

**Supplementary Figure 6 Calibration plots of DIVERT per country for outcome ED visits**


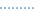

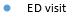


**Supplementary Figure 7 Calibration plots of CARS per country for outcome hospital admissions**
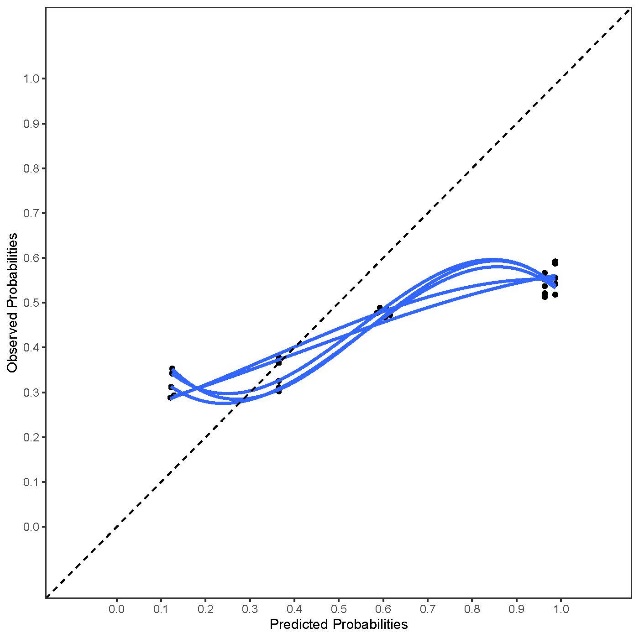

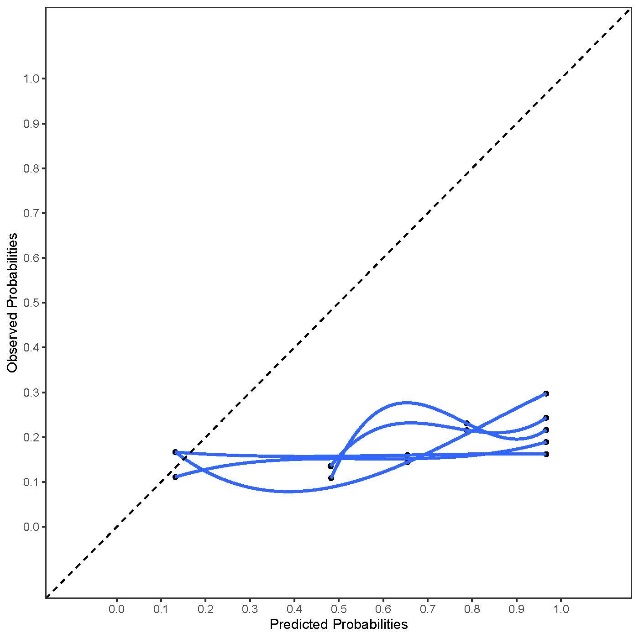


Italy

Iceland


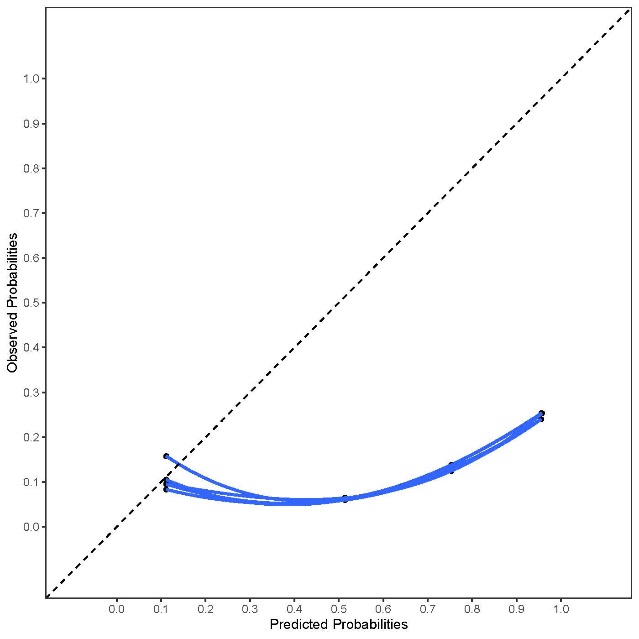


Germany


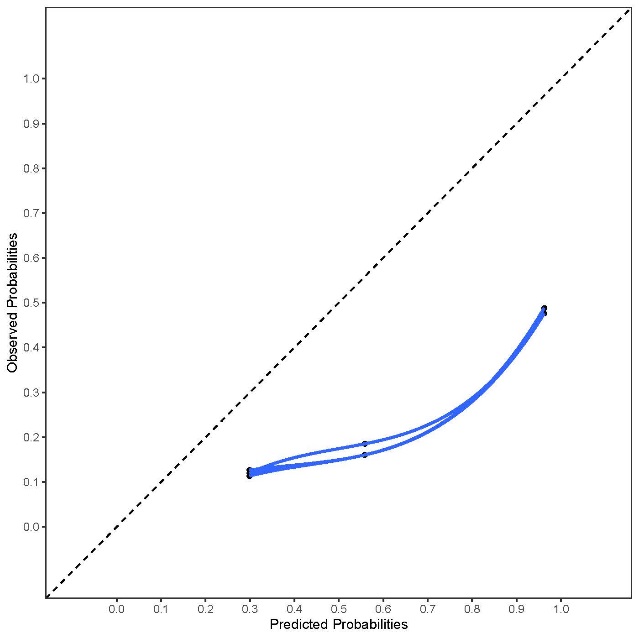

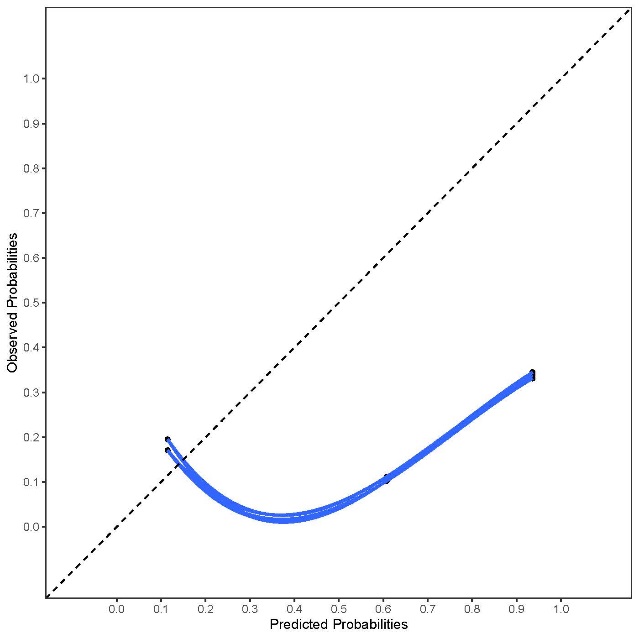


Finland

Netherlands

**Supplementary Figure 8 Calibration plots of CARS per country for outcome ED visits**


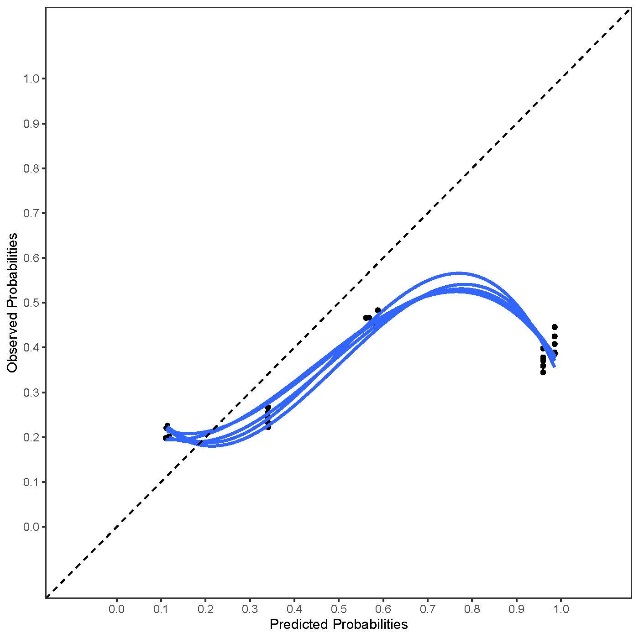

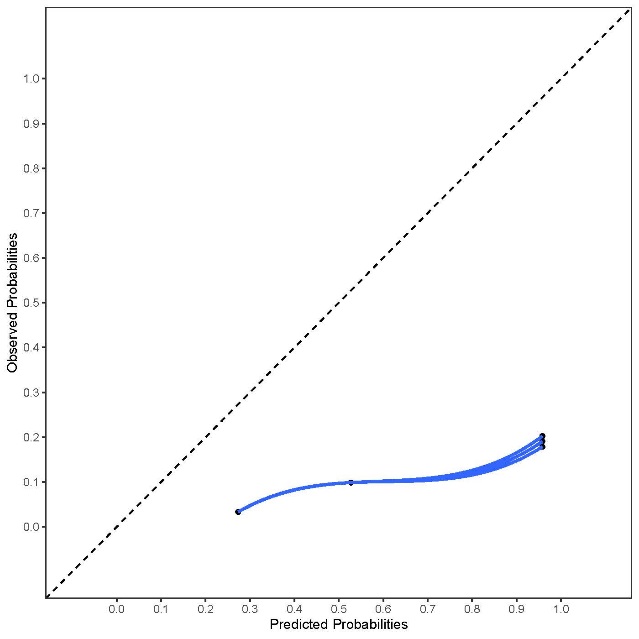


Iceland

Italy


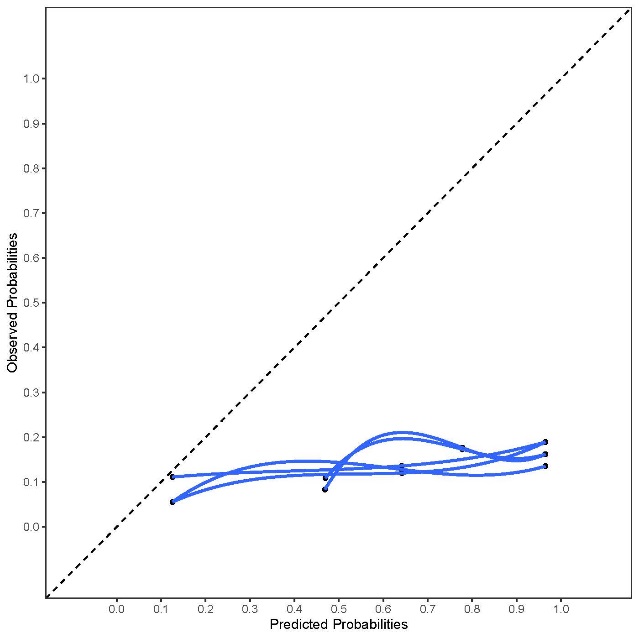

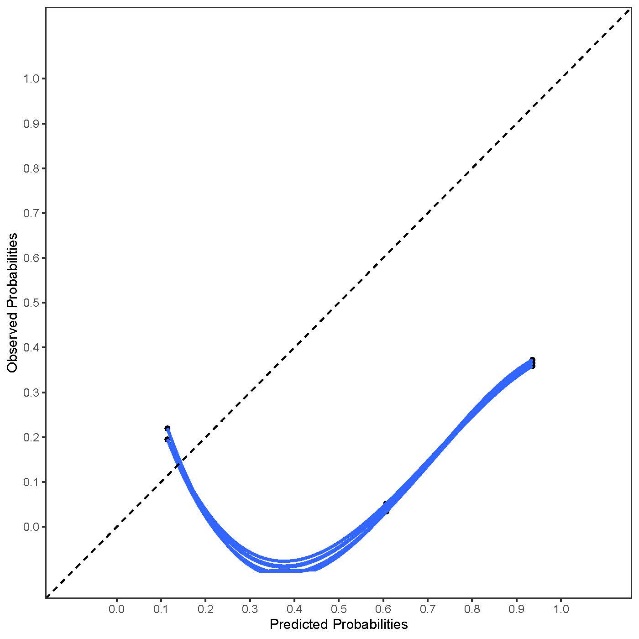


Finland

Netherlands

**Supplementary Figure 9 Calibration plots of CARS per country for outcome any unplanned hospital visit**


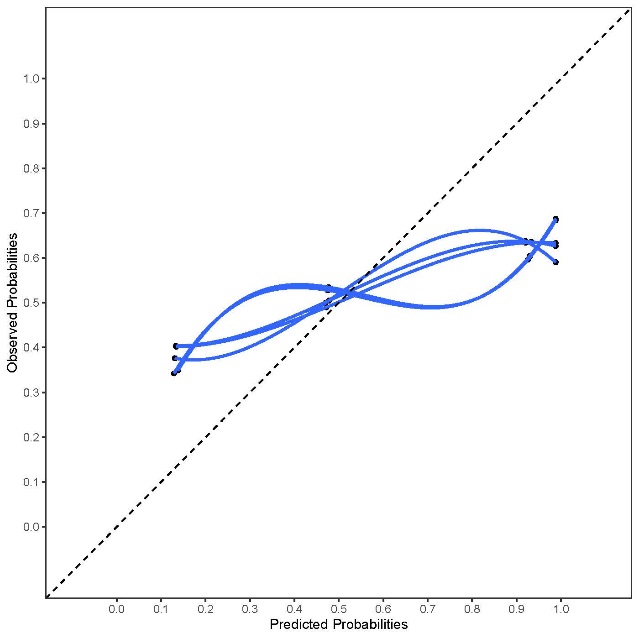

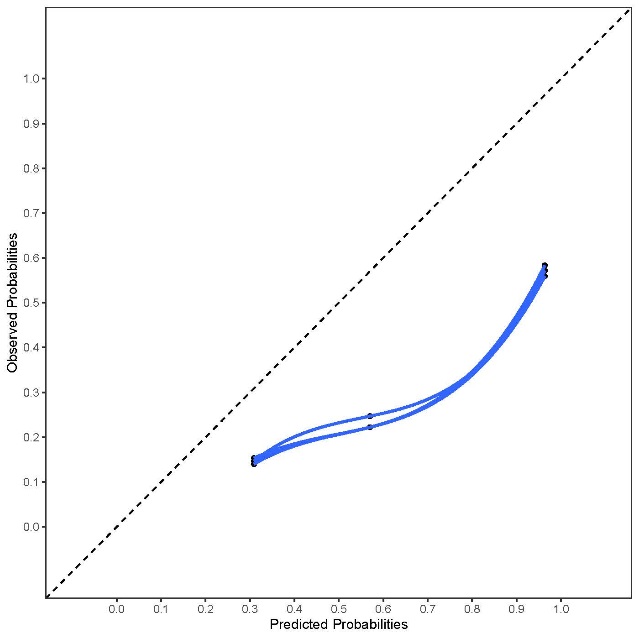


Finland

Iceland

Italy


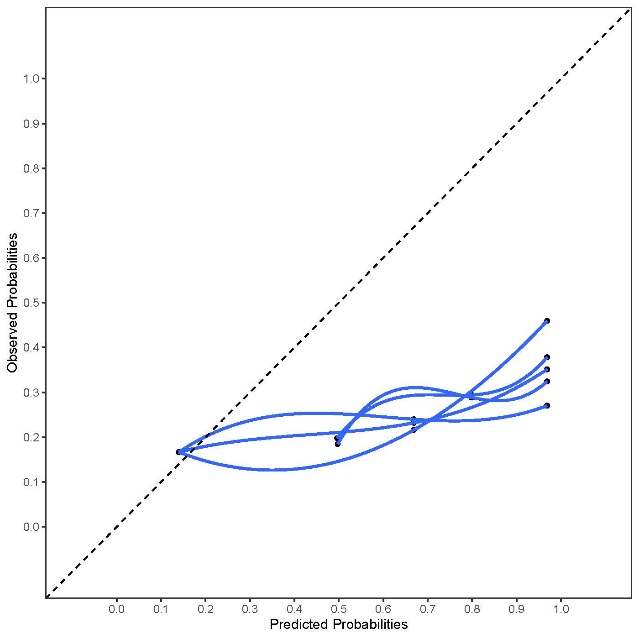

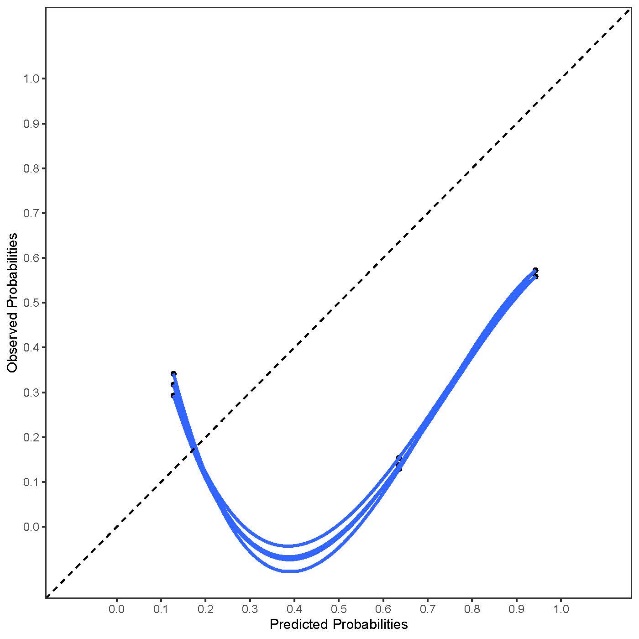


Netherlands

**Supplementary Figure 10 Calibration plots of EARLI per country for outcome hospital admissions**


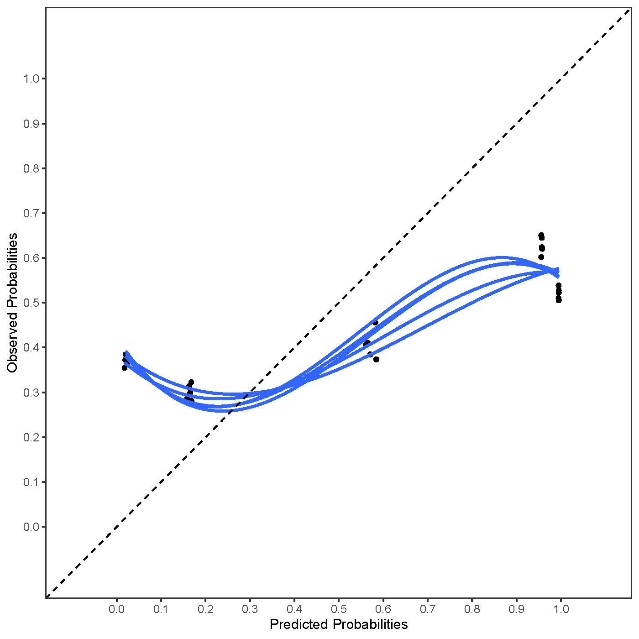

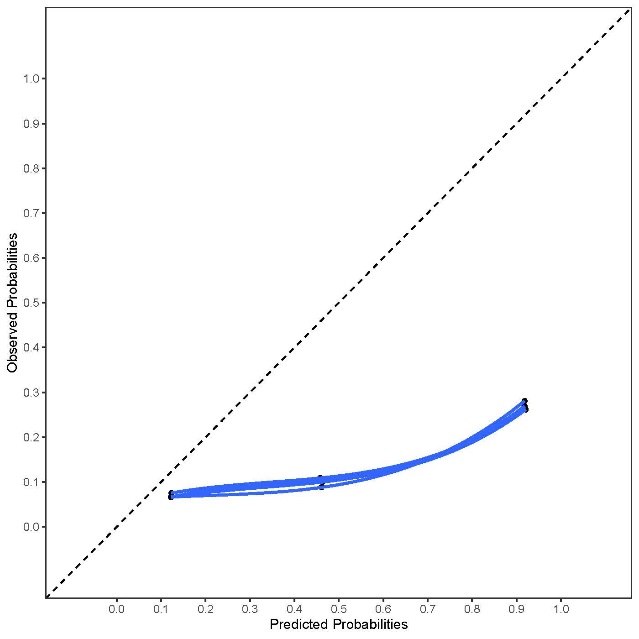

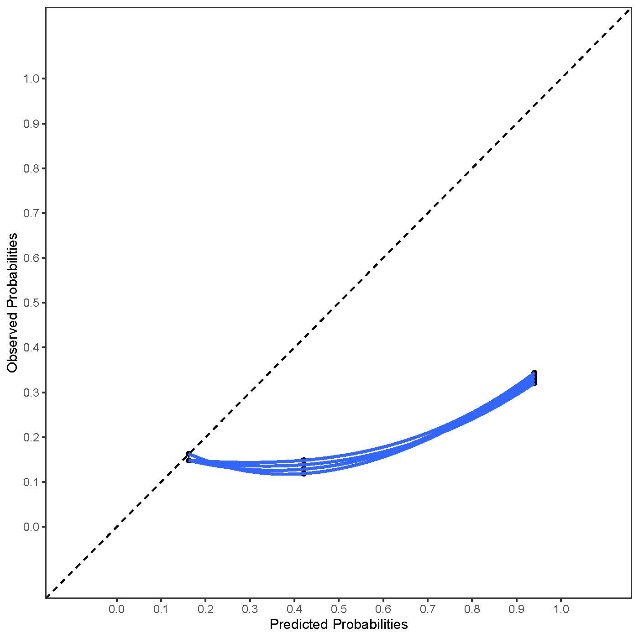


Finland

Iceland

Belgium

Netherlands

Italy


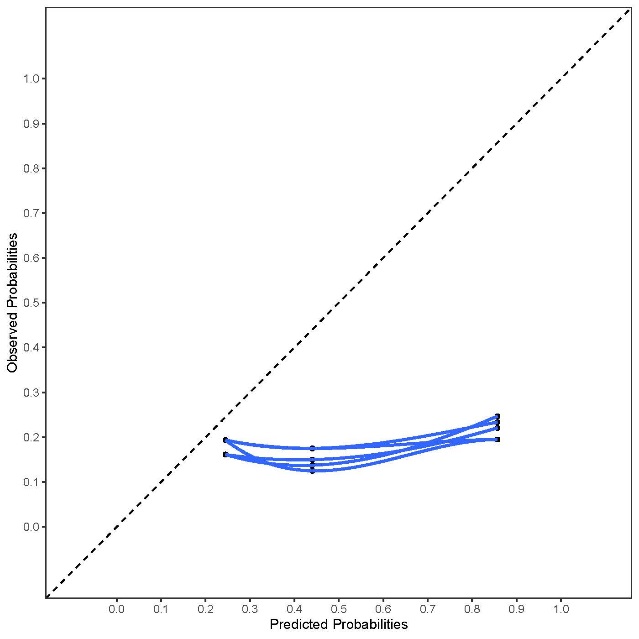

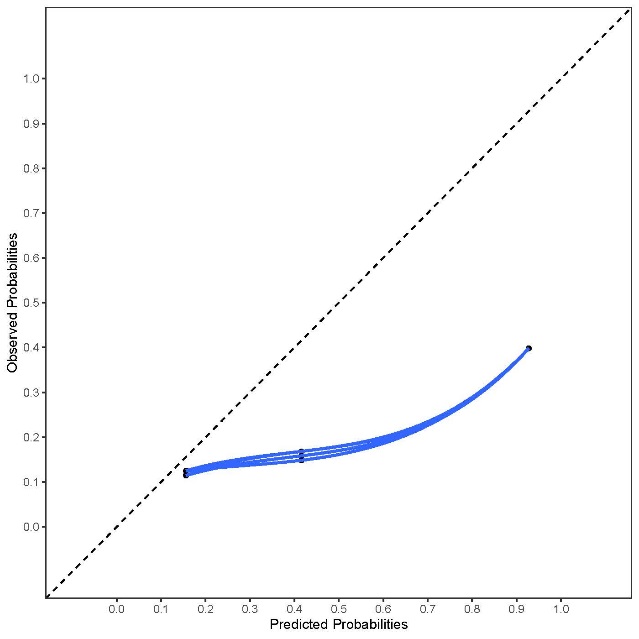


**Supplementary Figure 11 Calibration plots of EARLI per country for outcome ED visits**


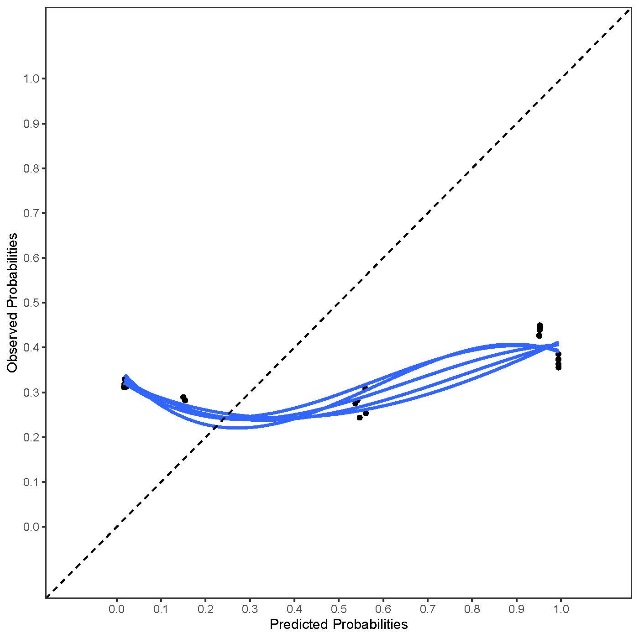

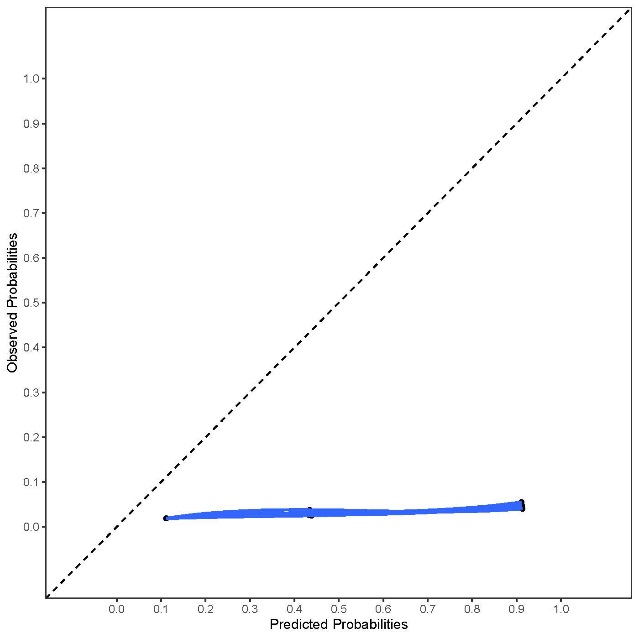

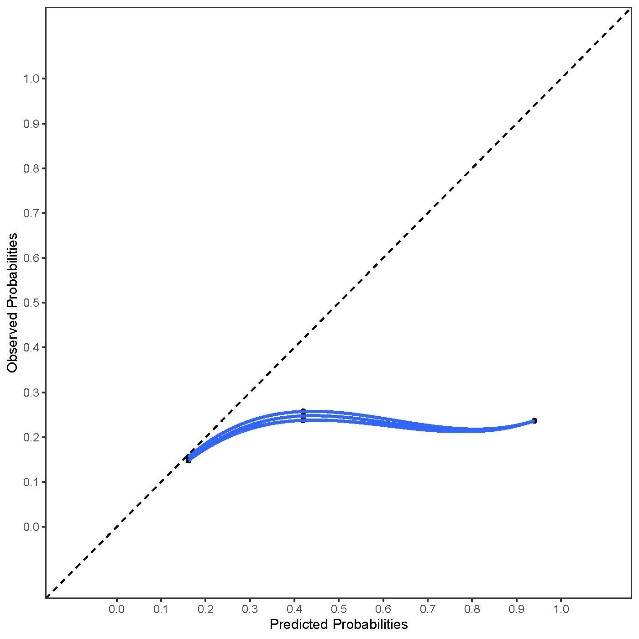


Finland

Iceland

Belgium

Netherlands

Italy


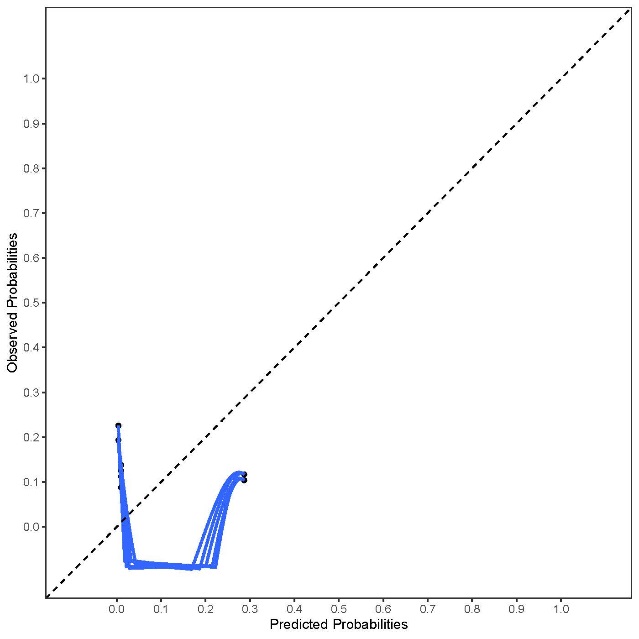

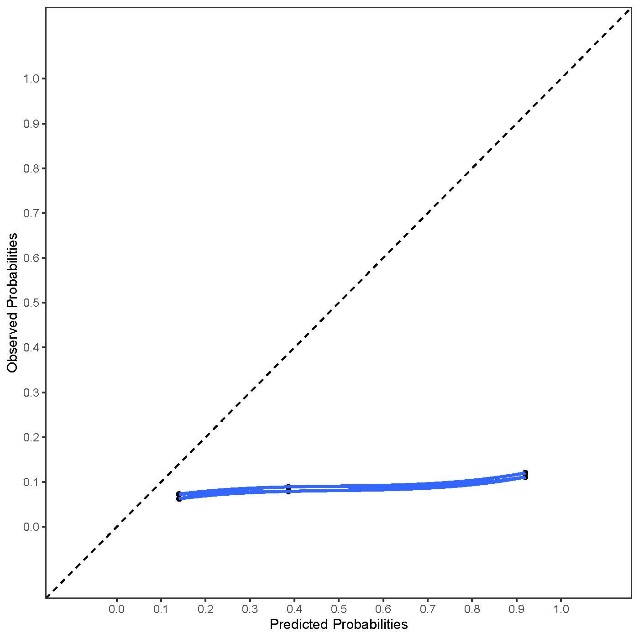


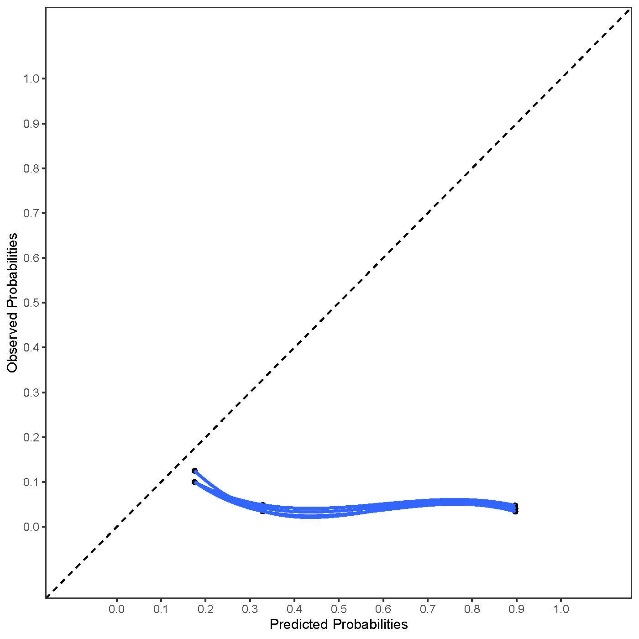


Germany

**Supplementary Figure 12 Calibration plots of EARLI per country for outcome any hospital visit**
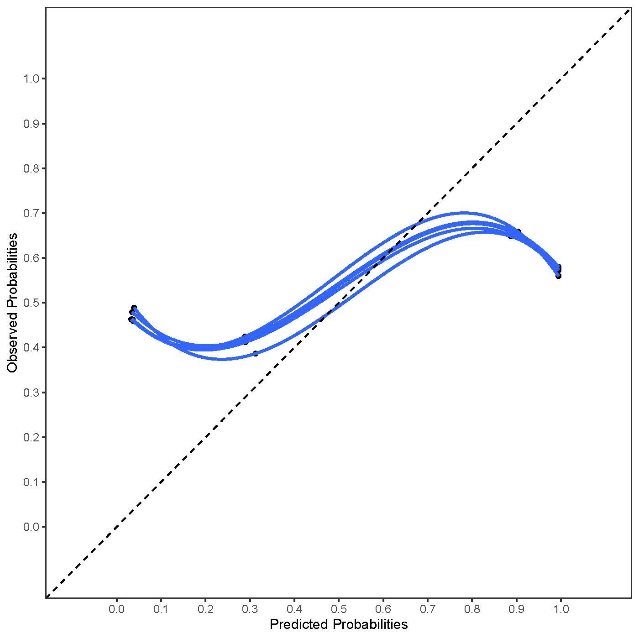

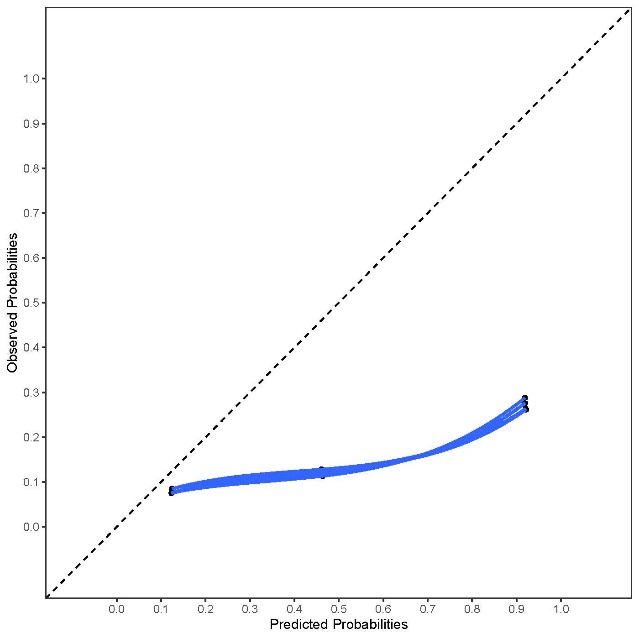


Italy


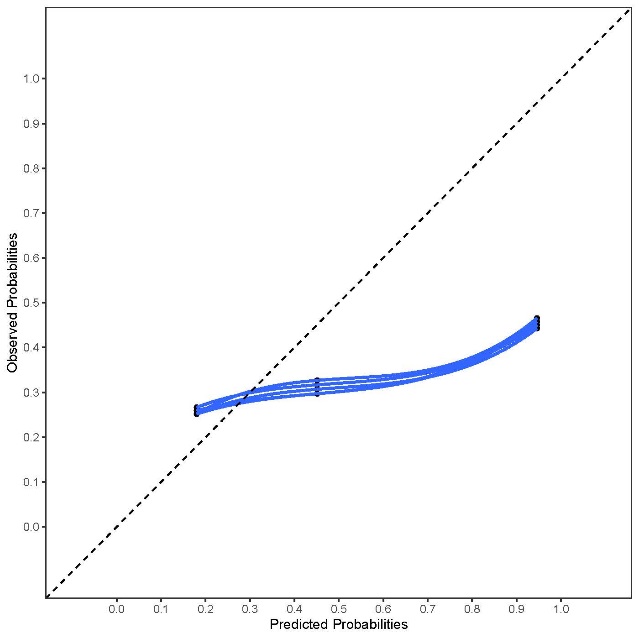


Finland


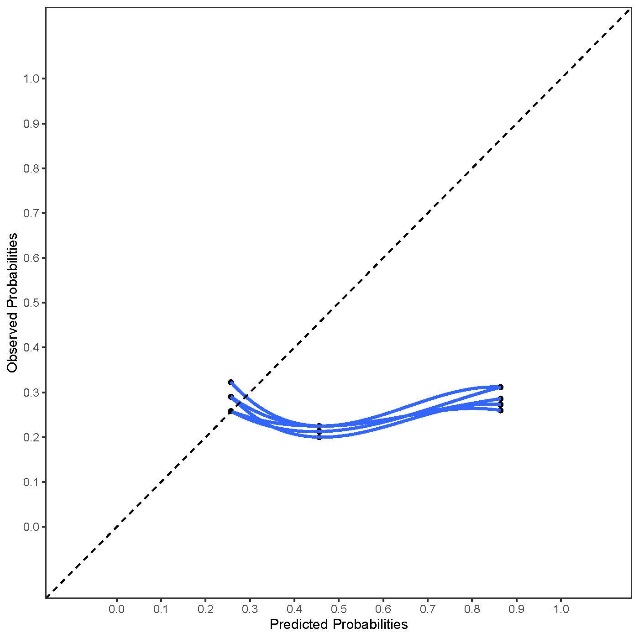


Belgium

Netherlands


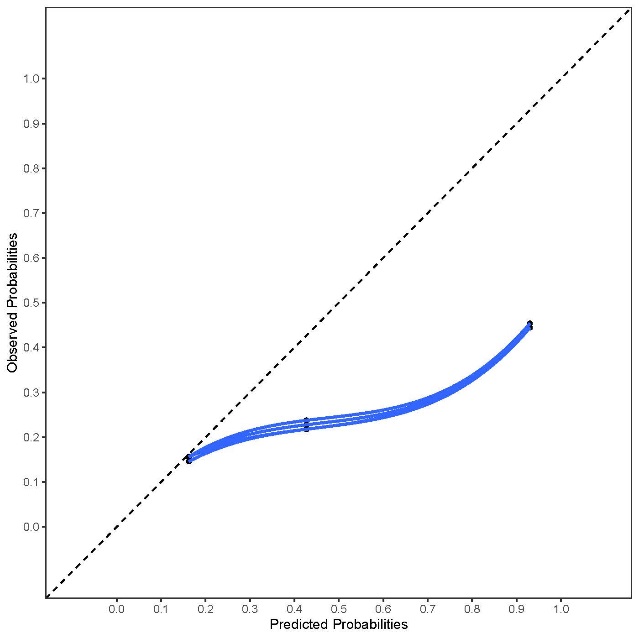


Iceland


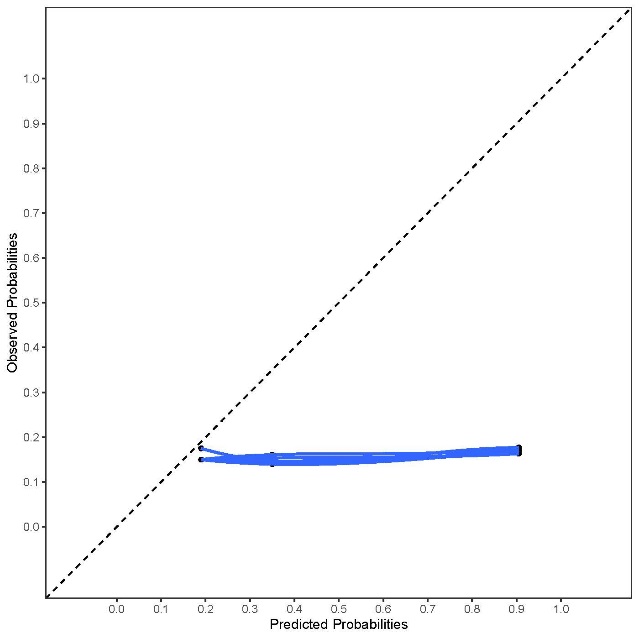


Germany

**Supplementary Figure 13 Calibration plots of PAA per country for all outcomes**


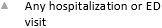

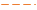

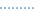

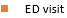

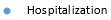


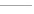


**Supplementary Figure 14 Calibration plots of CHESS per country for outcome hospital admissions**


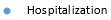

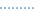

Supplement: Supplementary file 6 — Additional file 6. : Calibration plots. Calibration plots of the risk scores for each country, provided this could be assessed. [file 12877_2021_2521_MOESM6_ESM.docx]
